# Insights into gold-catalyzed formation of aza-heterocycles using benzofuroxans as nitrene transfer reagents: mechanism and origins of chemoselectivity†

**DOI:** 10.1039/d2ra05382a

**Published:** 2022-09-28

**Authors:** Weirong Wu, Jiehui Liang, Biaolin Jiang, Xiaoxuan Tian, Tingzhen Li

**Affiliations:** School of Environment and Chemical Engineering, Chongqing Three Gorges University Chongqing China wuweirong011@163.com litingzhen@163.com

## Abstract

Density functional theory (DFT) calculations have been performed to reveal the mechanism of gold(i)-catalyzed annulation of *N*-allylynamides and benzofuroxans as nitrene transfer reagents to construct azaheterocyclic compounds. The calculated results revealed that the reaction mechanism mainly undergoes eight processes. Among the reaction steps, intramolecular nucleophilic attack of the imino N atom on the α-position of activated gold keteniminium is a rate-determining process, which is different from that proposed previously by experiment. The chemoselectivity of the products is controlled by competition between the cyclopropanation of α-imino gold carbenes and intramolecular nucleophilic attack of the phenyl ring on α-imino gold carbenes, and could be explained by NPA charge. The different yields of cyclopropanated product in different solvents are dictated by the relative polarity leading to the different energy barriers of the rate-determining steps. The present work expounds the experimental observation at the molecular level and is informative for exploring efficient syntheses of 3-azabicyclo[3.1.0]hexanes.

## Abstract 2

DFT calculations were employed to study the mechanism and origin of chemoselectivity of gold-catalyzed annulation of *N*-allylynamides and benzofuroxans as nitrene transfer reagents.

## Graphical abstract

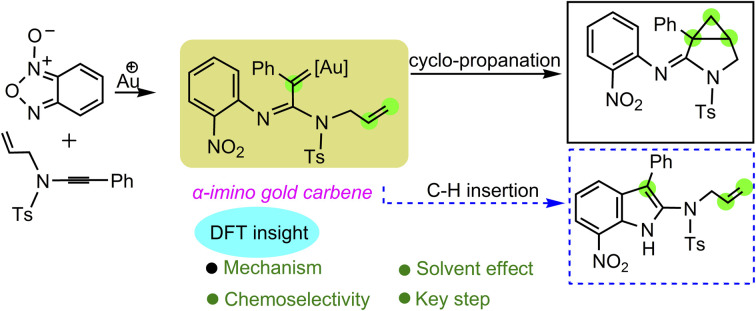

## 1. Introduction

**Scheme 1:**
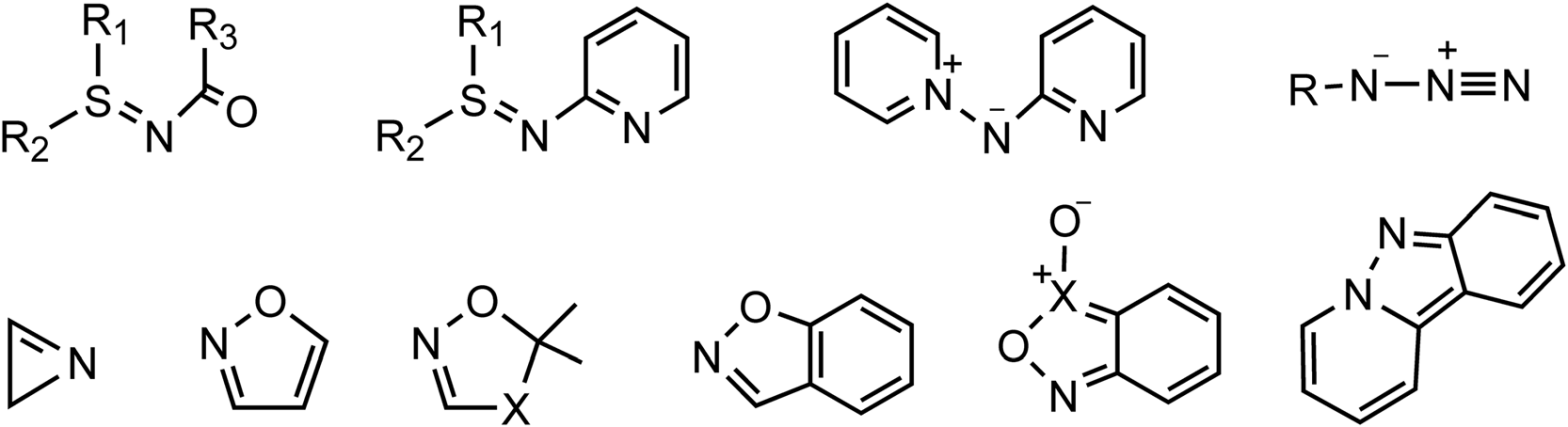
Some nitrene transfer reagents.

The cyclopropane core, a privileged motif in natural products and medicinal chemistry, has attracted tremendous attention ever since its discovery as it can serve as a versatile intermediate in organic reactions.^1^ Among the methods established for preparing cyclopropanes,^2^ gold-catalyzed reactions of nitrene transfer have been known as a promising methodology for the synthesis of bioactive miscellaneous aza-heterocyclic compounds.^3^ Considerable effort has been devoted to developing transfer reagents, such as triazapentalene,^4^ 2*H*-azirines,^5^ azides,^6^ isoxazole derivatives,^7^ anthranils,^8^ azirines,^9^ pyridine-based aza-ylides^10^ and sulfilimines,^11^ to trap α-imino gold carbenes as key electrophilic intermediates *en route* to functional materials and aza-heterocycles (Scheme 1). Among these synthetic strategies for the generation of α-imino gold carbenes, unsatisfactorily, many nitrene transfer reagents exhibit substantial drawbacks, such as azides with potential explosivity and ylides with poor reactivity. Thus, the search for and use of new nitrene transfer reagents is highly challenging and desired as such α-imino gold carbenes can effectively be used to construct diversely functionalized carbo- or heterocycles.

Recently, benzofurazan *N*-oxides^12^ have been considered as an effective and convenient nitrene transfer reagent for the generation of 2-amino-7-nitroindoles from ynamides due to its high reactivity, security, cheapness and accessibility.^13,14^ However, compared with other nucleophilic nitrenes, *N*-oxides used as the precursors of α-imino gold-carbene intermediates for the cyclopropanation are less explored, especially in gold chemistry.

**Scheme 2:**
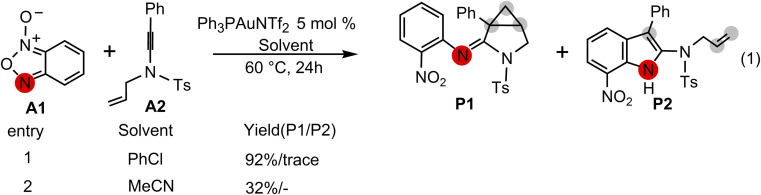
Gold-catalyzed annulation of benzofuroxan A1 with *N*-allylynamide A2 reported by Dubovtsev's group.^14^

Excitingly, Dubovtsev's group^14^ recently firstly reported a new approach for the generation of α-imino gold carbenes to construct 3-azabicyclo[3.1.0]hexanes *via* intramolecular cyclopropanation in highly selective gold-catalyzed annulations of *N*-allylynamides with benzofuroxans. In their representative one-pot reactions as indicated in Scheme 2, benzofuroxan A1 is treated with *N*-allylynamide A2 in the presence of Ph_3_PAuNTf_2_, the Gagosz catalyst, in chlorobenzene at 60 °C, leading to a 3-azabicyclo[3.1.0]hexane-2-imine P1 as the major product in 92% isolated yield. A only trace amount of indole P2 by CH insertion were detected. While the solvent chlorobenzene was replaced by acetonitrile, cyclopropanated product P1 with a yield of 32% was observed.

**Scheme 3:**
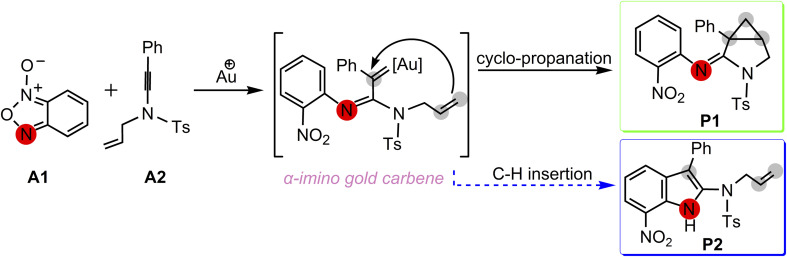
Postulated pathways proposed by Dubovtsev's group^14^ for the formation of 3-azabicyclo[3.1.0]hexane-2-imine P1*via* intramolecular cyclopropanation of the gold α-imino carbene intermediate.

A postulated reaction pathway for intramolecular cyclopropanation of gold α-imino carbenes generated from *N*-allylynamides and benzofuroxanes as nitrene transfer reagents was proposed by the Dubovtsev's group on the basis of their experimental observations (Scheme 3).

As for the uncommon reaction, although Dubovtsev's group proposed possible pathways, several important questions as to gold-catalyzed reaction of benzofuroxan A1 with *N*-allylynamides A2*via* intramolecular cyclopropanation of gold α-imino carbene intermediates remains still unclear. For instance, what is the actual detailed mechanism and the chemoselectivity of products for the reactions in Scheme 2 ? An explicit picture for the reaction routs is highly desired. Which one is a key step of the catalytic reaction? What is the solvent effect in the light of previous reports^15,16^? Intrigued by α-imino gold carbenes and chemoselectivities, we here performed a systematic theoretical study to provide a better understanding of the intrinsic mechanism for prototypical reactions in this field. Interestingly, the calculated energetic details discovered the characteristics of the gold-catalyzed nitrene transfer from benzofuroxan to *N*-allylynamides for the synthesis of aza-heterocycles, expounding the experimental phenomenon at the molecular level. Our calculated results are expected to be informative for the development of a new strategy to access fused *N*-heterocyclic compounds using *N*-oxides used as the precursors of α-imino gold-carbene intermediates.

## 2. Computational details

The calculations were conducted with the Gaussian 09 package.^17^ All structures were optimized at the B3LYP^18^/BSI level in the gas phase, BSI indicating a mixed basis set of the LANL2DZ^19^ augmented with a f-type polarization function for Au, P and S atom and the 6–31g (d) for C, H, O and N atom. The orbital exponents used in calculation are H(*ξ*_P_ = 0.11), O(*ξ*_d_ = 0.8), N(*ξ*_d_ = 0.8), P(*ξ*_d_ = 0.387), C(*ξ*_d_ = 0.8), S(*ξ*_d_ = 0.503), and Au(*ξ*_f_ = 1.050).^20^ Frequency calculations for all reported structures at the same level of theory were carried out to confirm all the stationary points as energy ninima or transition states. Intrinsic reaction coordinate (IRC) analysis^21^ was performed to confirm the connection of each transition state indeed with its forward and backward minima. To refine the calculated energies, we performed single-point free energy calculations using the solvation model density (SMD)^22^ with a larger combined basis set. The SDD^23^ (ECP) basis set is for Au, P and S, and the 6-311 + G(d, p) basis set for all the remaining atoms. Chlorobenzene and acetonitrile was used as the solvent with the UAKS atomic radii, in accordance with the experimental temperature. To reveal the detailed bonding feature, natural bond orbital (NBO) analyses were employed for the selected species.

## 3. Results and discussion

### 3.1 Formation of α-imino gold carbene intermediates

**Scheme 4:**
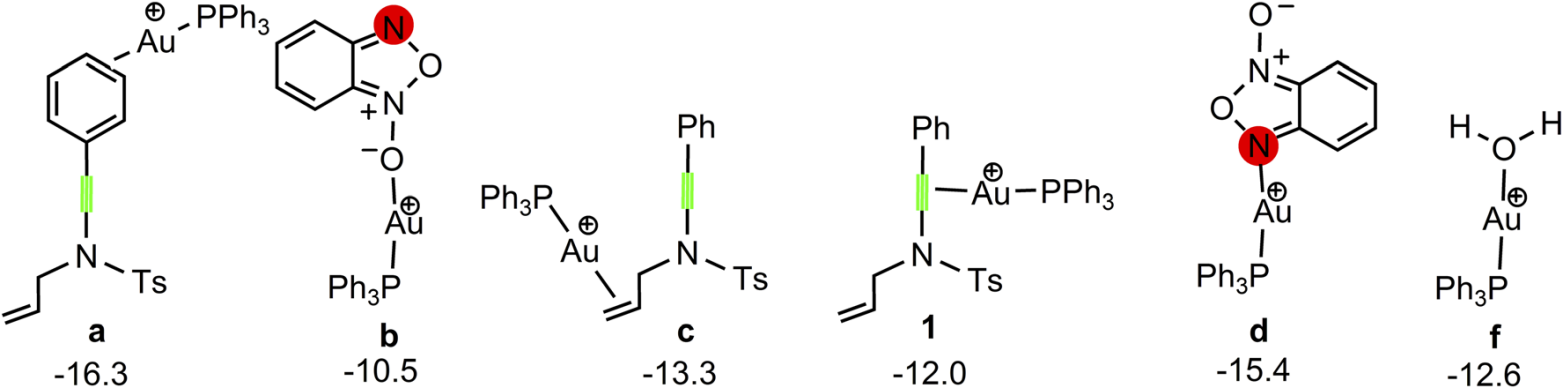
Various potential coordinated gold(i) complex. The Gibbs free energies are given in kcal mol^−1^.

**Fig. 1:**
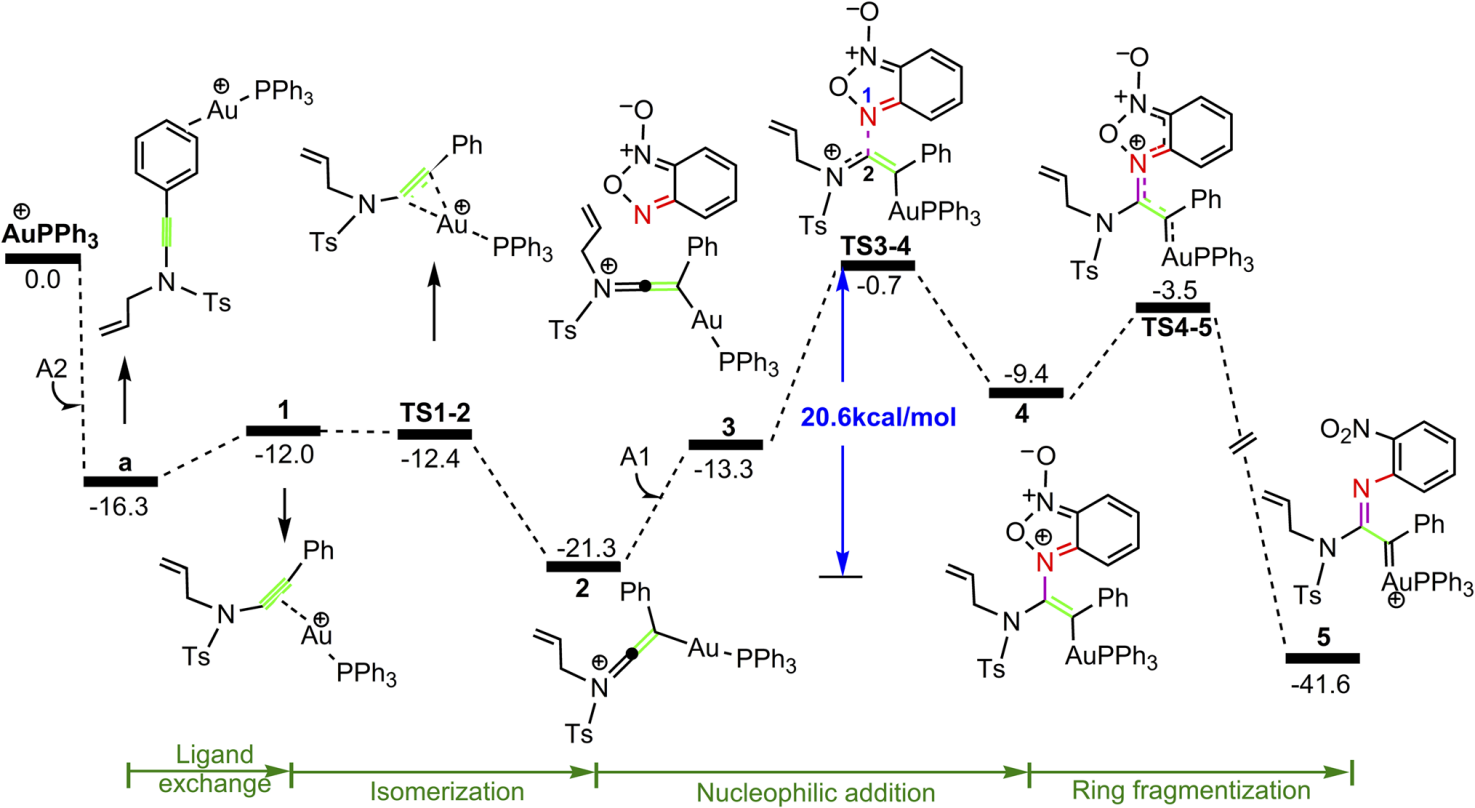
Potential energy surface in PhCl solvent for the formation of gold α-imino carbene intermediate 5. The Gibbs free energies are in kcal mol^−1^.

Our attention at first focuses on the reaction of benzofuroxan A1 with *N*-allylynamide A2 (eqn (1) in Scheme 2). To establish the most stable gold species, we investigated various potential coordinated complexes in the reaction system. All of the possible cationic PPh_3_Au^+^ adducts formed with H_2_O molecular, substrates A1 and A2 are shown in Scheme 4. Clearly, adduct a is likely the resting state of the catalyst due to the fact that gold(i) complex is coordinated to the phenyl C=C π bond of *N*-allylynamide A2 more strongly than other substrate. Fig. 1 shows the calculated formation pathways of α-imino gold carbene species.

**Fig. 2:**
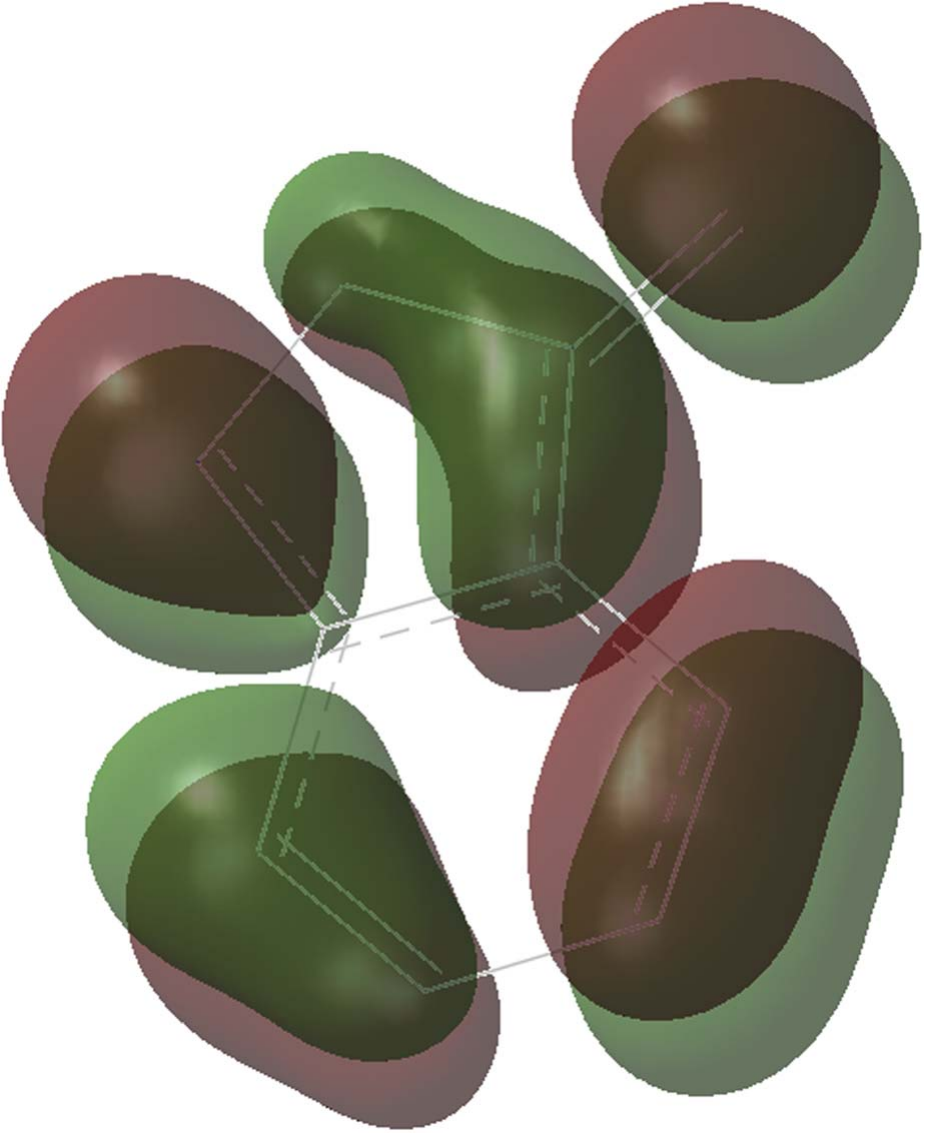
The HOMO molecule orbital of benzofuroxan A1.

As shown in Fig. 1, the coordination of cationic PPh_3_Au^+^ species with phenyl C=C π bond of *N*-allylynamide A2 affords initially a more stable gold(i) complex a as the resting state of the catalyst, releasing an energy of 16.3 kcal mol^−1^. Subsequently, ligand exchange between alkynyl triple bond and phenyl C=C π bond of *N*-allylynamide A2 takes place to give the alkyne-coordinated gold(i) complex 1, from which isomerization process occurs *via*TS1-2, leading to the formation of a gold keteniminium species 2 without much of a kinetic barrier. This result implies that in agreement with previous proposals that interaction of gold complexes with electron-rich ynamides would facilely produce a electrophilic keteniminium species.^24^ To facilitate subsequent nucleophilic attack, a unstable complex 3 relative to 2 is generated with the participating of benzofuroxan A1. In the following step, the reaction is surmised to proceed with nucleophilic attack of the imino N1 atom from the benzofuroxan A1 on α-position of gold activated keteniminium species 2 to afford the vinyl-gold intermediate 4*via*TS3-4 with an overall barrier of 20.6 kcal mol^−1^ (the difference between TS3-4 and 2), and this process is identified as the rate-determining step for the catalytic reaction, which is different from that proposed by Dubovtsev's group.^14^ In addition, we also considered the possibility of intramolecular attack by the O1 atom of N^+^–O^−^ moiety instead of the imino N1 atom in A1 on *in situ* generated ketenimine intermediate 2 to trigger generation of highly electrophilic α-oxo gold carbene intermediates. Unfortunately, the tying was failed, which is consistent with the literature that the oxygen transfer reaction did not occur.^25^ In nucleophilic reagent A1, obviously, the O1 atom of N^+^–O^−^ moiety is much less reactive than the imino N1 atom in A1 as a nucleophile. The fact can be rationalized by the highest occupied molecular orbital (HOMO) of A1. As shown in Fig. 2, electron density of the imino N1 atom is larger than that of the O1 atom of N^+^–O^−^ moiety according to HOMO molecule orbital of A1. And the basicity of the imino N1 atom is stronger than the O1 atom of N^+^–O^−^ moiety in A1. Henceforth, the reactivity of the imino N1 atom is better, which coincides with experimental observation^25^ and previously theoretical calculation.^26^ Next, ring fragmentization of 4 occurs to produce the reactive α-imino gold carbene 5, a key intermediate. The low barrier (5.7 kcal mol^−1^) implies that this process is quite facile.

### 3.2 Formation of final product P from 5

**Fig. 3:**
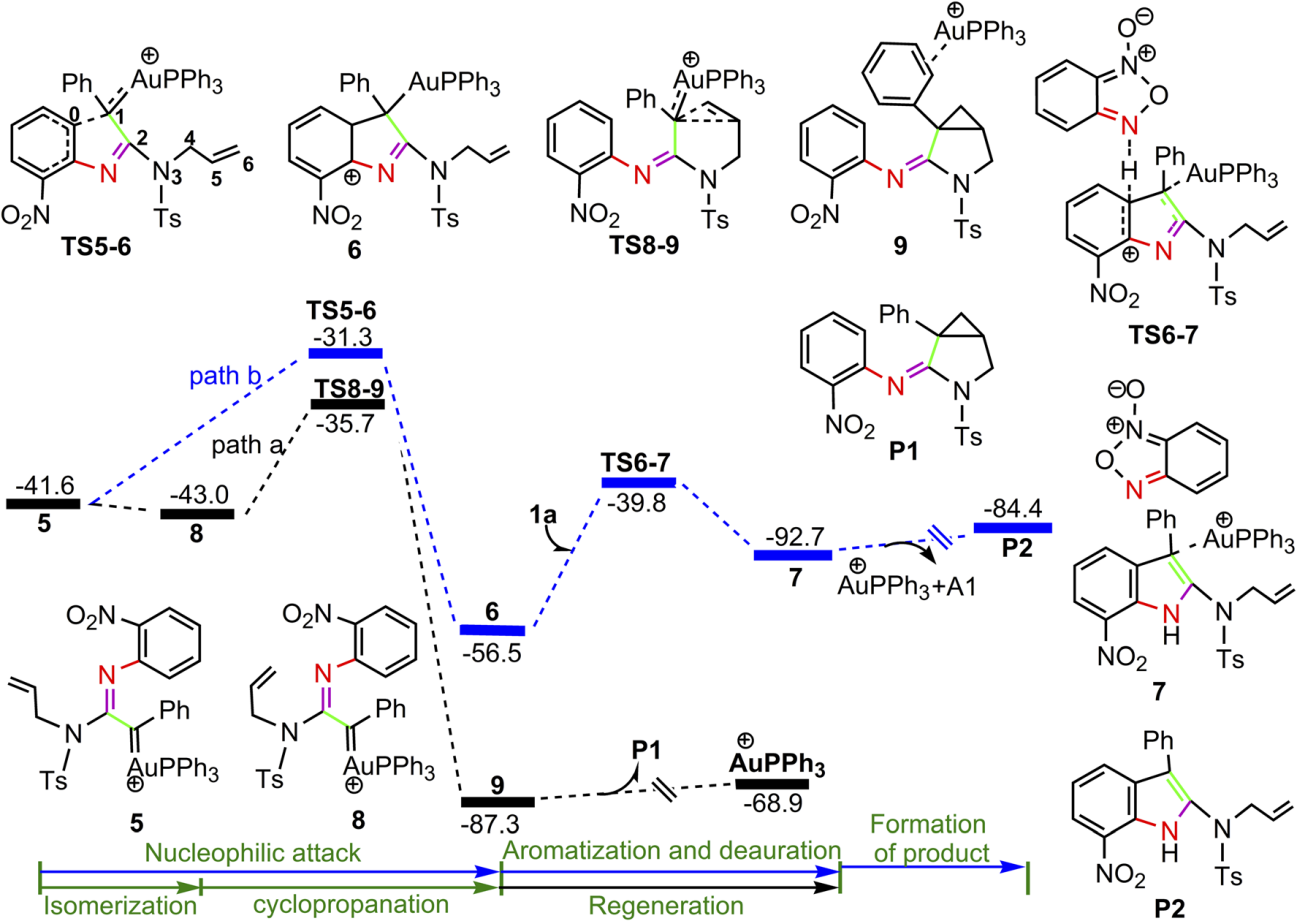
Potential energy surface in PhCl solvent for the transformation of 5 to P. The solvent-corrected relative free energies are given in kcal mol^−1^.

**Fig. 4:**
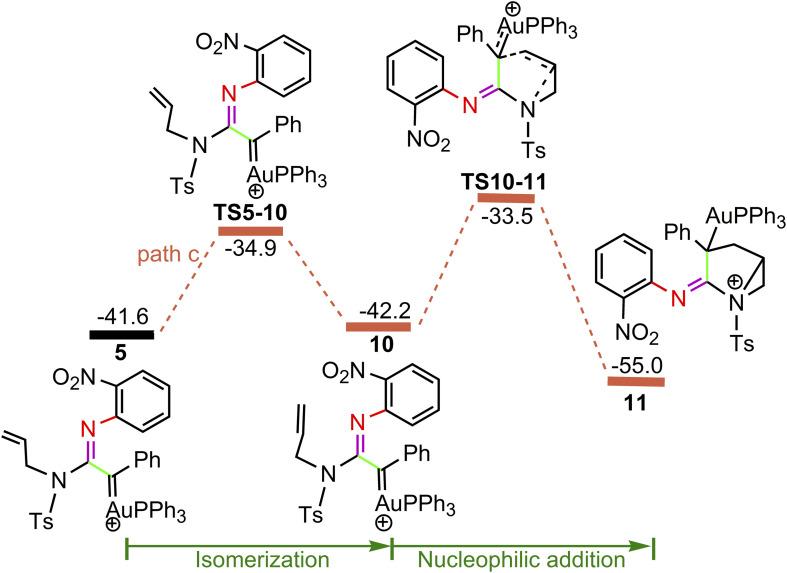
Potential energy surface in PhCl solvent for the other pathway of nucleophilic attacks on α-imino gold carbene 5. The solvent-corrected relative free energies are given in kcal mol^−1^.

The calculated profiles for the transformation of α-imino gold carbene intermediate 5 into cyclopropane product P1 and indole P2 are collected in Fig. 3. Once the gold α-imino carbene intermediate 5 is formed, there are two possibilities of nucleophilic attacks on gold carbenes and one possibility of cyclopropanation of gold carbenes (path a and b in Fig. 3, and path c in Fig. 4). In Fig. 3, path a starts from the rotation of the N3–C4–C5–C6 dihedral angle in 5 (D(N3C4C5C6) = −127.9°), leading to a slightly more stable isomer 8 (D(N3C4C5C6) = −3.4°) ready for subsequent intramolecular cyclopropanation. Then, concurrent additions of alkenyl C5 and C6 atoms to the gold carbene C1 atom take place *via* transition structure TS8-9 with a low barrier of 7.3 kcal mol^−1^ to generate product-coordinated complex 9. Finally, the desired product P1 is gained along with release of cationic PPh_3_Au^+^ species. In contrast, intramolecular attack of the phenyl moiety on the Au-carbene C1 is relatively energy demanding with an activation barrier of 10.3 kcal mol^−1^*via* transition structure TS5-6 to generate a extremely unstable intermediate 6 over 8 by 30.8 kcal mol^−1^ (path b). The obvious free difference between TS5-6 (31.3 kcal mol^−1^) and TS8-9 (35.7 kcal mol^−1^) might be a result of de-aromatization transition state TS5-6. Subsequently, 6 would undergo aromatization by substrate A1-assisted H-shift with simultaneous deauration process to give complex 7, the precursors of product P2, *via* transition state TS6-7 with an activation barrier of 16.7 kcal mol^−1^. The ensuing concomitant dissociation of A1 molecule and cationic PPh_3_Au^+^ catalyst lead to the product P2.

For the path c of nucleophilic addition starting from 5 shown in Fig. 4, a isomerization process *via*TS5-10 affords a slightly stable intermediate 10 by the slight rotation of C5=C6 π bond around C4–C5 bond in 5 (5: D(N3C4C5C6) = −127.9°, 10: D(N3C4C5C6) = −129.8°). Subsequently, 10 would undergo intramolecular nucleophilic attack on the Au-carbene to give intermediate 11. However, transition state TS10-11 (−33.5 kcal mol^−1^) has a higher free energy than TS8-9 (−35.7 kcal mol^−1^) in Fig. 3, which indicated that the pathway towards intermediate 11 would be unfavorable. Based on Fig. 1 and 3, it is clear that the key step of the whole catalytic cycle is intramolecular nucleophilic addition of the imino N1 atom to Au-carbene C1 *via*TS3-4 (2→3→TS3-4→4) rather than intramolecular cyclopropanation of α-imino gold carbene intermediates as proposed previously by the experiments, and the overall barrier for this step is calculated to be as much as 20.6 kcal mol^−1^, which is comparable with experimental conditions (60 °C), indicating that the pathway *via* the cyclopropanation of α-imino gold carbenes appears to be more favorable in the experimental condition (60 °C).

### 3.3 Origin of chemoselectivity

On the basis of the model reaction calculated (eqn (1) in Scheme 1), among the three pathways calculated, path a leading to the formation of cyclopropane product P1*via*TS8-9 (−35.7 kcal mol^−1^) is the most favourable pathway than path b towards indole P2*via*TS5-6 (−31.3 kcal mol^−1^) and path c *via*TS10-11 (−33.5 kcal mol^−1^) to give 11. Experimentally, the reaction of benzofuroxan A1 and *N*-allylynamide A2 catalyzed by Ph_3_PAuNTf_2_ gave a 3-azabicyclo[3.1.0]hexane-2-imine P1 (92% yield) and P2 (trace). Clearly, TS8-9 and TS5-6 are the excellent chemodivergence-determining transition states towards two products (P1 and P2). The overall barrier for 8→TS8-9 associated with the cyclopropanation of α-imino gold carbene 8, another isomer of 5, is lower by 3.0 kcal mol^−1^ in comparison to 5→TS5-6 related to intramolecular nucleophilic attack of the phenyl ring on gold carbene in 5, explaining the experimentally observed results of the two products. The C–H insertion mechanism starting from 5 (path b) is unfavorable because the phenyl ring with the electron-withdrawing substituent (–NO_2_ group) is not nucleophilic enough to make facilely the nucleophilic attack on the gold carbene, which might be corroborated by the NBO charge population analysis, the charge carried by phenyl C0 atom, and alkenyl C5 and C6 atoms in 5 are computed as −0.259*e*, −0.242*e* and −0.409*e*, respectively.

### 3.4 Effect of solvent

**Fig. 5:**
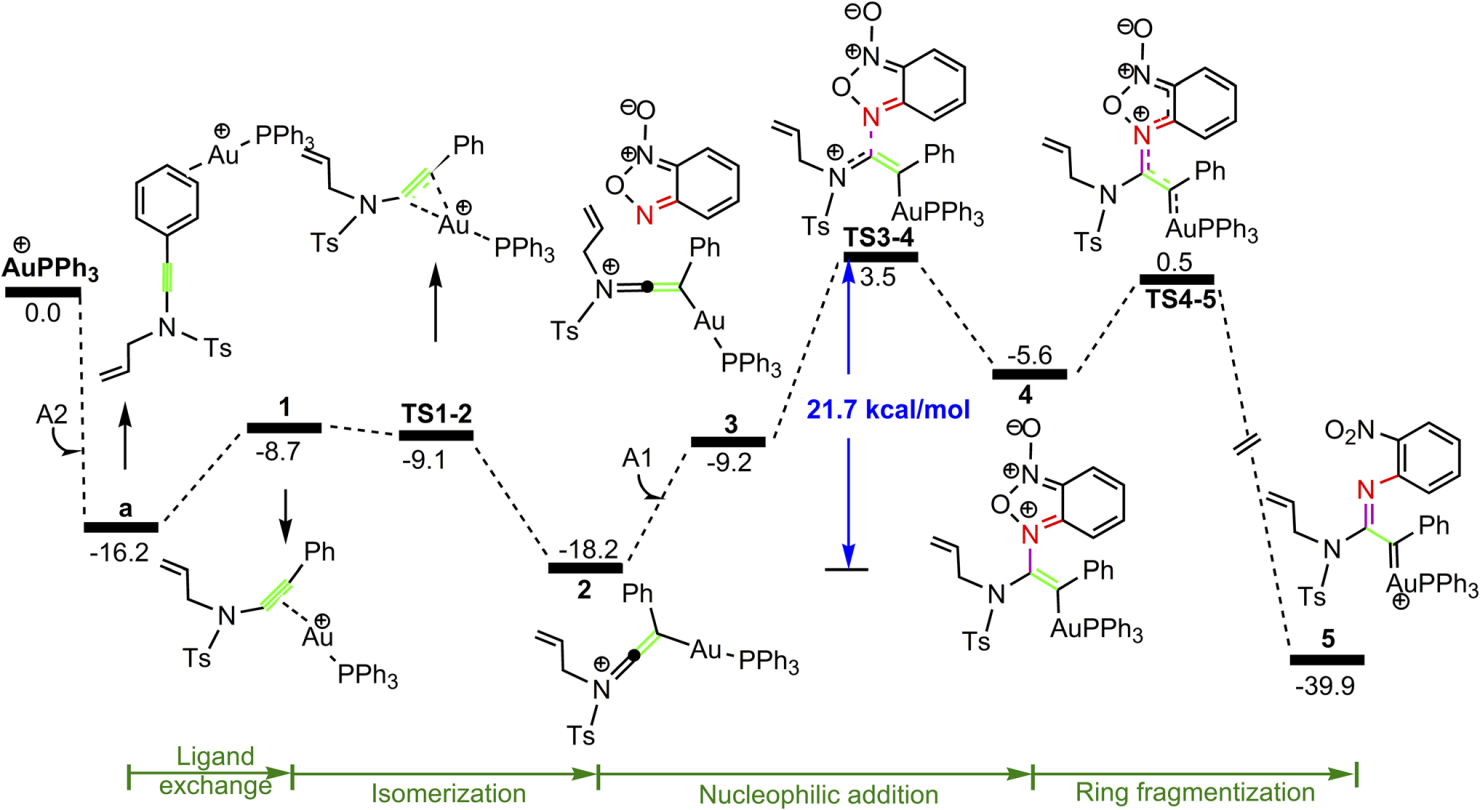
Potential energy surface in MeCN solvent for the formation of the gold α-imino carbene intermediate 5. The Gibbs free energies are in kcal mol^−1^.

Experiments showed that the yield of major product P1 in acetonitrile (MeCN) decreases to 32%. It is obvious that solvent effects are involved in the reactivity of the catalytic reaction. In order to address this question, we calculated the relative free energy profiles towards P1 and P2 considering acetonitrile as the solvent. Fig. 5, S1 and S2† shows the potential energy surface in MeCN solvent for the reaction paths leading to P1 and P2. As compared with the Gibbs energy profiles in PhCl, the obvious difference is the energy barrier of the rate-determining step (from 2 to 4) of the whole catalytic cycle. For comparison, the calculated formation pathways of the α-imino gold carbene intermediate 5 in MeCN solvent are exhibited Fig. 5. Different form the situation in PhCl, the energy barrier calculated for nucleophilic attack by the imino nitrogen atom is 21.7 kcal mol^−1^ when MeCN is used as the solvent, higher than the overall barrier (20.6 kcal mol^−1^) for the same process in PhCl, implying the nucleophilic addition is achievable and almost consistent with the experimental fact that the yield of P1 reached 32%. A plausible explanation for the solvent effects might be given as followed. As discussed above, almost all of the transition states and intermediates for the pathways are cationic species, and thus have relatively higher dipole moment. Clearly, the relative polarity of the TS3-4 and 2 is the key factor leading to the different energy barriers of the rate-determining steps in the two solvents. The barrier differences ΔΔ*G* = (Δ*G*(TS3-4) − Δ*G*(2)) calculated in the PhCl and MeCN are 20.6 and 21.7 kcal mol^−1^, respectively, indicating that the energy gap increases when the polarity of the solvent used increases. As a result, the strong polarity of MeCN as a solvent influences TS3-4 and 2 more strongly than those in PhCl, resulting in the decrease of P1 yield.

### 3.5 Proposed mechanism based on calculation

**Scheme 5:**
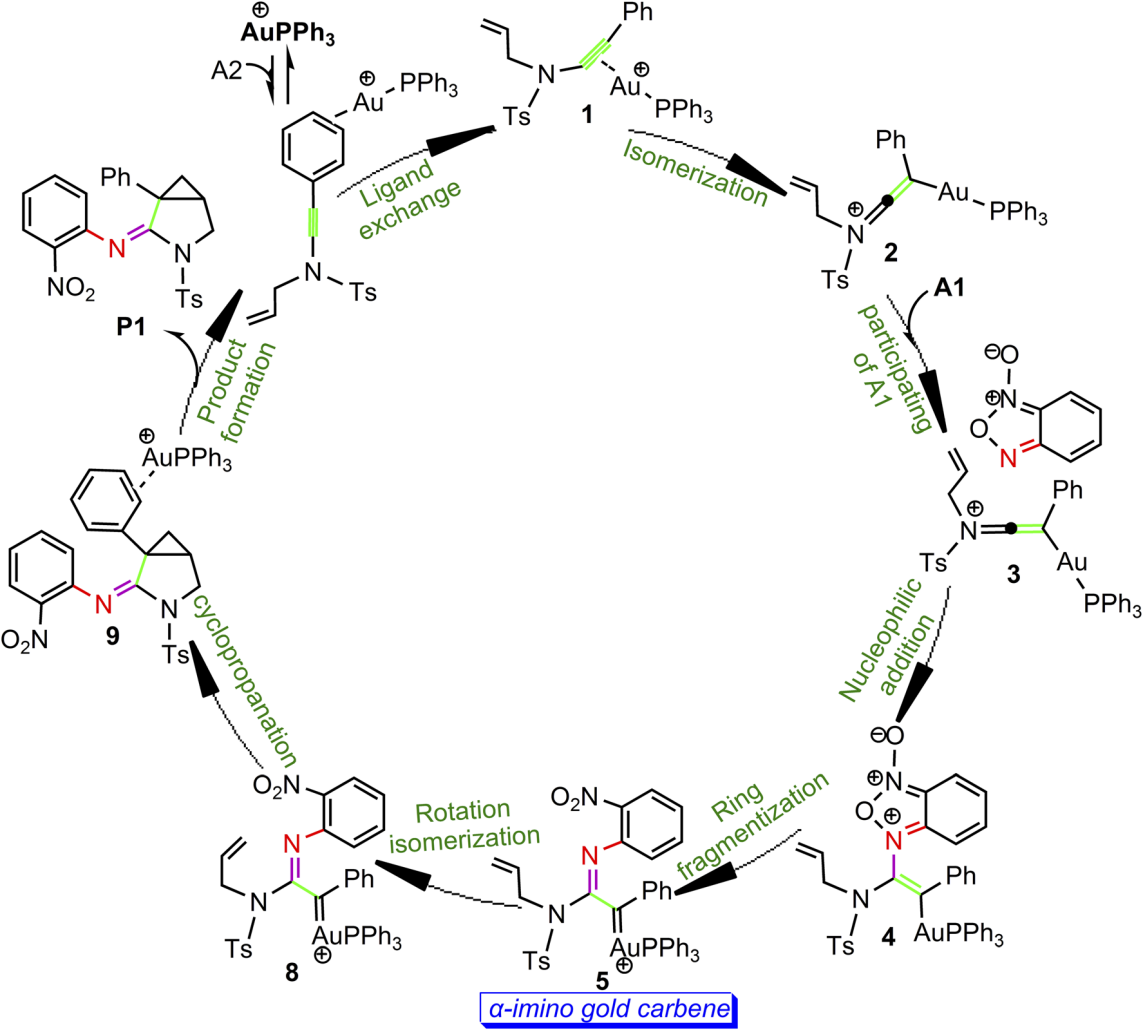
Catalytic cycle derived from DFT calculations.

According to the above calculation results, the gold-catalyzed annulation of benzofuroxan A1 as nitrene transfer reagents with *N*-allylynamide A2 are summarized schematically in Scheme 5. In this catalytic reaction, complex a is predicted to be the catalytic resting state due to the fact that gold(i) complex is coordinated to the phenyl C=C π bond of *N*-allylynamide A2 more strongly than other substrate. Ligand exchange between phenyl C=C π bond and alkyne occurs to give a alkyne-coordinated species 1. The activation of *N*-allylynamide with Au(i) catalyst subsequently generates a highly electrophilic keteniminium intermediate 2. With the participating of benzofuroxan A1, a unstable complex 3 relative to 2 is generated. Next, the nucleophilic attack of the imino N1 atom in benzofuroxan A1 to α-position of activated gold keteniminium 2 to produce vinyl-gold intermediate 4. The formation of α-imino gold carbene intermediate 5, a key intermediate, occurs with ring fragmentization. Subsequent isomerization of 5 by the rotation of alkenyl C=C bond afford α-imino Au-carbene intermediate 8. Finally, 8 undergoes the cyclopropanation process to give the desired product 3-azabicyclo[3.1.0]hexane-2-imine P1. According to our calculations, the transformation 2 + A2→4 with an overall activation energy of 20.6 kcal mol^−1^ is the rate-determining step, completely different from that proposed by Dubovtsev's group.

## 4. Conclusion

The detailed mechanisms of the gold(i)-catalyzed annulation of benzofuroxan A1 with *N*-allylynamide A2 have been investigated with the aid of DFT calculations. The reaction pathways calculated mainly involve eight processes: ligand exchange between phenyl C=C π bond and alkyne, *N*-allylynamide activation to produce gold keteniminium species (isomerization), participating of benzofuroxan A1, intramolecular nucleophilic addition of imino N atom to α-position of the activated gold keteniminium, ring fragmentization of the vinyl-gold intermediate, isomerization by the rotation of alkenyl C=C bond, and cyclopropanation of the α-imino gold carbene leading to product formation. The chemoselectivity is controlled by competition of the cyclopropanation of α-imino gold carbenes and the nucleophilic attack of the phenyl ring on α-imino gold carbenes. The key step of the whole catalytic cycle is intramolecular nucleophilic addition of the imino N1 atom to Au-carbene C1 rather than intramolecular cyclopropanation of α-imino gold carbene complexes as proposed previously by the experiments, and the overall barrier for this step is calculated to be as much as 20.6 kcal mol^−1^. The solvent effects could be revealed by the relative polarity leading to the different energy barriers of the rate-determining steps.

## Conflicts of interest

The authors declare no competing financial interest.

## Supplementary Material

RA-012-D2RA05382A-s001
