# Supplementary material for: Insights into gold-catalyzed formation of aza-heterocycles using benzofuroxans as nitrene transfer reagents: mechanism and origins of chemoselectivity
Source: RSC Adv. 2022 Sep 28;12(42):27483–91. doi: 10.1039/d2ra05382a (PMC9516356; doi:10.1039/d2ra05382a)
Supplement: RA-012-D2RA05382A-s001 [file RA-012-D2RA05382A-s001.pdf]

Electronic Supplementary Material (ESI) for RSC Advances.
This journal is © The Royal Society of Chemistry 2022

Supporting Information

Insights into Gold-Catalyzed Formations of Aza-Heterocycles Using Benzofuroxans as Nitrene Transfer Reagents: Mechanism and Origins of Chemoselectivity

Weirong Wu, * Jiehui Liang, Biaolin Jiang, Xiaoxuan Tian, and Tingzhen Li*

School of Environment and Chemical Engineering, Chongqing Three Gorges University, Chongqing, China

**Correspondent author: email: wuweirong011@163.com*

Fig. S1 Potential energy surface in MeCN solvent for the transformation of **5** to **P**. The solvent-corrected relative free energies are given in kcal mol^{-1}.

Fig. S2 Potential energy surface in MeCN solvent for the other pathway of nucleophilic attacks on α-imino gold carbene **5**. The solvent-corrected relative free energies are given in kcal mol^{-1}

Cartesian coordinates and electronic energies for all of the calculated structure

A1

SCF done: -490.829084

C	0.23003900	-0.93663000	0.00005700
C	-0.17523500	0.42967700	-0.00003400
C	0.73970900	1.51110100	0.00005700
C	2.06875900	1.17729200	-0.00000700
C	2.50312000	-0.19569700	-0.00005200
C	1.62520200	-1.24640000	-0.00000500
H	0.38374600	2.53526000	0.00026700
H	2.82033700	1.96055500	-0.00007500
H	3.57083500	-0.39503300	-0.00012100
H	1.95342200	-2.27992500	0.00013200
O	-1.92085200	-0.94930100	-0.00002900
N	-0.81287900	-1.75536600	0.00003200
N	-1.51857800	0.44222700	-0.00002000
O	-2.37386000	1.31618500	-0.00001900

A2

SCF done: -911.293127

C	-5.14874900	1.07798900	-0.95058000
C	-3.90477900	0.45859900	-1.03999000
C	-3.29275700	-0.08166900	0.10999700
C	-3.96781300	0.01100900	1.34392700
C	-5.21809800	0.61999100	1.41996400
C	-5.81263800	1.15950500	0.27645200
H	-5.60578200	1.49236700	-1.84535200
H	-3.39303300	0.38353900	-1.99459300
H	-3.50240300	-0.40028600	2.23468400
H	-5.72807700	0.67853900	2.37795700
H	-6.78586600	1.63824400	0.34063900
C	-2.01448800	-0.70564800	0.01937000
C	-0.92814700	-1.23786300	-0.10264400
S	1.40032600	-1.20855500	-1.36980000
O	2.52487400	-2.16177200	-1.34575300
O	0.61312700	-0.91936700	-2.57674100
C	1.95137400	0.35263900	-0.68131600
C	3.12020500	0.39531500	0.07873900
C	1.20726000	1.50938400	-0.93322800
C	3.53926200	1.61749600	0.60274100
H	3.69232900	-0.51031500	0.24462900
C	1.63984800	2.71767600	-0.39468600
H	0.31763900	1.45849200	-1.55054800
C	2.80790700	2.79227600	0.38102100

H	4.45311900	1.65919700	1.18984800
H	1.06611500	3.62099300	-0.58737000
C	3.25837700	4.11246400	0.95994600
H	4.23713900	4.02672100	1.44135400
H	3.32936500	4.88376400	0.18395300
H	2.54689300	4.47738500	1.71154200
N	0.26344100	-1.84992400	-0.21246300
C	0.73322200	-2.75799100	0.86291000
H	-0.10748300	-3.43510700	1.05712900
H	1.55280800	-3.34807100	0.44978000
C	1.14876700	-2.04865800	2.12374800
C	2.30572000	-2.26415600	2.75031600
H	0.42727500	-1.33916100	2.52837600
H	2.55759100	-1.75392800	3.67610400
H	3.04222800	-2.96704800	2.36573100

a

SCF done: -1748.065031

C	-1.11188300	-3.35886900	-1.35297800
C	0.21921700	-2.95741300	-1.29551300
C	0.91595100	-2.94764400	-0.06281500
C	0.24720600	-3.38987200	1.10767200
C	-1.08298100	-3.77837700	1.05735500
C	-1.80706900	-3.73677700	-0.16821100
H	-1.62267100	-3.40586600	-2.31043300
H	0.74027900	-2.65335900	-2.19710100
H	0.78762800	-3.40612000	2.04813900
H	-1.58226300	-4.12527600	1.95726900
H	-2.77872400	-4.22078600	-0.23904600
C	2.23882800	-2.45986700	-0.01337600
C	3.35825800	-1.97845200	-0.01654300
S	4.90031600	-0.34512000	-1.41836400
O	5.87003600	-1.03384500	-2.28486300
O	3.56678200	0.06873500	-1.88789300
C	5.71842500	1.02068800	-0.60670300
C	7.07821200	1.23251100	-0.84156100
C	4.97723400	1.87892900	0.21212400
C	7.70372800	2.31952400	-0.23204900
H	7.62728000	0.56203600	-1.49382200
C	5.62317900	2.95445200	0.81243200
H	3.91836300	1.70530400	0.37481200
C	6.99210600	3.19492600	0.60016100
H	8.76217900	2.48984300	-0.40903500
H	5.05847100	3.62305400	1.45703000

C	7.67114100	4.38479200	1.23256000
H	8.74062300	4.20576700	1.37849400
H	7.57232300	5.27225200	0.59374500
H	7.22831200	4.63095100	2.20287600
N	4.56078700	-1.40701500	-0.03325000
C	5.72479000	-2.07161000	0.62117700
H	5.97673200	-2.99061800	0.08118800
H	6.56577300	-1.37784400	0.51670300
Au	-2.47318100	-1.50643300	-0.00204500
P	-3.06080000	0.74198500	0.06621200
C	-4.18140600	1.16398000	-1.32471900
C	-3.97149200	2.30082500	-2.11815000
C	-5.27658000	0.32291900	-1.58588400
C	-4.85404000	2.59264400	-3.16059400
H	-3.12456500	2.95290600	-1.93133700
C	-6.15555000	0.62405700	-2.62444700
H	-5.44254600	-0.56408200	-0.97909500
C	-5.94363200	1.75830100	-3.41389500
H	-4.68553500	3.47206000	-3.77507700
H	-7.00094700	-0.02855500	-2.82126300
H	-6.62557000	1.98754400	-4.22746600
C	-3.91061000	1.22594100	1.61802000
C	-3.45197400	0.70037400	2.83685500
C	-4.98448300	2.12847700	1.60813500
C	-4.05749300	1.08160700	4.03277800
H	-2.62448500	-0.00473800	2.85026700
C	-5.58810400	2.50317000	2.81041400
H	-5.35357800	2.53189400	0.67051500
C	-5.12635300	1.98238900	4.02028400
H	-3.70054800	0.67121100	4.97277700
H	-6.42167500	3.19913900	2.79819100
H	-5.60147800	2.27278500	4.95272400
C	-1.55558500	1.78474800	-0.08659200
C	-1.51608400	3.07617000	0.46355700
C	-0.44035100	1.29187900	-0.78263600
C	-0.37491200	3.86338000	0.30398900
H	-2.36563000	3.46388400	1.01754100
C	0.70073900	2.08025700	-0.93541400
H	-0.45655200	0.29025000	-1.20298800
C	0.72993500	3.36799200	-0.39359800
H	-0.34935700	4.86245000	0.72932700
H	1.56188500	1.68043600	-1.46370500
H	1.61571500	3.98562400	-0.51303400
C	5.44917100	-2.34356900	2.07194500

H	5.21765800	−1.47258700	2.68347000
C	5.50309100	−3.55928600	2.61545700
H	5.73230000	−4.44227000	2.02285500
H	5.33500300	−3.71609200	3.67705600

b

SCF done: −1327.603731

Au	−0.44433800	−0.47560000	−0.00699400
P	1.78240800	0.03110500	0.00209700
C	2.74543700	−1.23319800	−0.91054400
C	3.97859100	−1.69324700	−0.42558000
C	2.24635100	−1.72243500	−2.12921200
C	4.70347100	−2.63559400	−1.15772300
H	4.37047300	−1.32649300	0.51765000
C	2.97823900	−2.65987900	−2.85551700
H	1.28967600	−1.37247300	−2.50918400
C	4.20593900	−3.11862700	−2.36921000
H	5.65579500	−2.99279900	−0.77727800
H	2.58793000	−3.03637900	−3.79631100
H	4.77135400	−3.85463100	−2.93315300
C	2.09200000	1.65418700	−0.79009900
C	1.23610700	2.72953400	−0.49896200
C	3.18108700	1.84203800	−1.65384200
C	1.47257700	3.98073200	−1.06521600
H	0.38830400	2.59013700	0.16716100
C	3.40806400	3.09791700	−2.22028000
H	3.84503300	1.01659800	−1.88952200
C	2.55710000	4.16506300	−1.92774600
H	0.80785000	4.80889700	−0.83835200
H	4.24973200	3.23886500	−2.89182100
H	2.73564600	5.13923400	−2.37336100
C	2.43420400	0.10890000	1.71308500
C	3.34168500	1.10796800	2.09526100
C	2.03761600	−0.86896700	2.64036100
C	3.84672200	1.12428900	3.39685500
H	3.64846700	1.87144200	1.38763800
C	2.54978200	−0.84764800	3.93626900
H	1.33186500	−1.64365700	2.35102500
C	3.45269400	0.14992000	4.31554800
H	4.54596600	1.90123900	3.69118800
H	2.24031700	−1.60475400	4.65054100
H	3.84534800	0.16862800	5.32794900
C	−5.35712600	0.92633500	−0.00332800
C	−4.82365000	−0.40402000	−0.00147500

C	-5.63819200	-1.56369500	0.00857700
C	-6.98880700	-1.33315400	0.01653700
C	-7.54851300	-0.00336300	0.01479800
C	-6.77142500	1.12216200	0.00514300
H	-5.20378600	-2.55624000	0.00971100
H	-7.67127000	-2.17705700	0.02445800
H	-8.62935800	0.09496200	0.02145300
H	-7.18543200	2.12379300	0.00374500
O	-3.21721600	1.11604700	-0.01798000
N	-4.38456100	1.82714300	-0.01292600
N	-3.50066400	-0.25441500	-0.01054200
O	-2.54533900	-1.09653700	-0.01457000

c

SCF done: -1748.065772

C	-6.48832800	4.79211900	0.91537200
C	-5.62338000	3.71465300	1.08891300
C	-5.36219500	2.83320500	0.02057700
C	-5.98869900	3.05935900	-1.22144200
C	-6.85194900	4.13927400	-1.38745900
C	-7.10337900	5.00712300	-0.32111200
H	-6.68554000	5.46432300	1.74554200
H	-5.14624800	3.53882800	2.04811800
H	-5.79190700	2.37935600	-2.04471600
H	-7.33114000	4.30441600	-2.34827900
H	-7.77832800	5.84807700	-0.45288200
C	-4.47733700	1.72483500	0.19485500
C	-3.73283200	0.77923600	0.36482700
S	-3.48073000	-1.66706700	1.44466100
O	-2.35184300	-2.61792500	1.35812500
O	-3.99169400	-1.17953500	2.73477600
C	-4.84047200	-2.22945500	0.44064300
C	-4.58740300	-3.09058800	-0.63067300
C	-6.13690600	-1.82926300	0.76889000
C	-5.66060700	-3.54321400	-1.39108300
H	-3.57349300	-3.40798500	-0.84916200
C	-7.19551600	-2.29353100	-0.00903600
H	-6.30777500	-1.17537700	1.61650400
C	-6.97805700	-3.15486900	-1.09435400
H	-5.47673800	-4.21633900	-2.22435000
H	-8.20835000	-1.98544000	0.23555400
C	-8.13528700	-3.68184200	-1.90685800
H	-7.85856400	-3.82006900	-2.95704300
H	-8.46344400	-4.65772300	-1.52568500

H	-8.99627100	-3.00799000	-1.86392300
N	-2.89156200	-0.27077000	0.49666800
C	-1.45014800	0.01218100	0.62430600
H	-1.27027100	0.85931000	1.29704500
H	-0.98213500	-0.88308700	1.04210900
Au	1.47747000	0.51609000	-0.55597900
P	3.70656000	-0.00921500	-0.03466200
C	4.68202100	1.51424200	0.26305900
C	5.98695100	1.64903000	-0.23308200
C	4.11141000	2.55014900	1.02171000
C	6.71200000	2.81285400	0.03087400
H	6.43483900	0.85647300	-0.82382800
C	4.84364700	3.70632300	1.28465000
H	3.10012300	2.45250100	1.40939700
C	6.14329400	3.83919300	0.78705100
H	7.72096600	2.91558200	-0.35723500
H	4.39911900	4.50430700	1.87182500
H	6.71003400	4.74374400	0.98696300
C	3.79176900	-1.03995100	1.47881700
C	2.85511800	-2.07272300	1.65168200
C	4.78269600	-0.82545100	2.44820400
C	2.91221700	-2.88386100	2.78347200
H	2.08494500	-2.24617300	0.90383700
C	4.83177900	-1.64136100	3.58026400
H	5.50687100	-0.02622500	2.32651700
C	3.89947100	-2.66677000	3.74912700
H	2.18357400	-3.67817700	2.91433400
H	5.59755700	-1.47091700	4.33110800
H	3.93831300	-3.29466800	4.63432500
C	4.51009900	-0.93553200	-1.39674600
C	5.37857700	-2.00468900	-1.13110100
C	4.26704100	-0.54518000	-2.72415400
C	5.99771300	-2.67413300	-2.18845700
H	5.56799800	-2.31788200	-0.10934200
C	4.89265100	-1.21569300	-3.77364700
H	3.59430700	0.28211700	-2.93788000
C	5.75628200	-2.28189200	-3.50630100
H	6.66648600	-3.50377900	-1.97962300
H	4.70221200	-0.91060600	-4.79827100
H	6.23730700	-2.80790900	-4.32563600
C	-0.91538700	0.28697300	-0.76040000
H	-1.00752100	-0.53511000	-1.46966700
C	-0.53527100	1.52412000	-1.21286200
H	-0.59955900	2.39891400	-0.56878300

H	-0.35451000	1.70357500	-2.27022000

d

SCF done: -1327.611285

Au	0.63618300	-0.19660700	0.01284000
P	-1.66071400	0.03697000	0.00461300
C	-2.45280700	-1.34333100	-0.90567400
C	-3.55662200	-1.12215200	-1.74278400
C	-1.95645200	-2.64608000	-0.73272800
C	-4.15665700	-2.19894000	-2.39838800
H	-3.94322300	-0.11859400	-1.88927600
C	-2.56317400	-3.71588600	-1.38845600
H	-1.09995400	-2.82548900	-0.08735200
C	-3.66214500	-3.49263600	-2.22273700
H	-5.00874800	-2.02386500	-3.04842400
H	-2.17503000	-4.72092400	-1.25293600
H	-4.12956500	-4.32664900	-2.73787500
C	-2.34902000	0.04945300	1.70387200
C	-1.64715800	0.71190900	2.72417700
C	-3.57693300	-0.56684600	1.98858400
C	-2.17239500	0.76052700	4.01446300
H	-0.69295400	1.18772100	2.51154700
C	-4.09482600	-0.51633700	3.28391100
H	-4.12329100	-1.08863200	1.20928900
C	-3.39537000	0.14501100	4.29518600
H	-1.62510900	1.27198500	4.80066000
H	-5.04334500	-0.99814500	3.50160200
H	-3.79979500	0.17741000	5.30256300
C	-2.16153700	1.60103500	-0.81317000
C	-3.16329100	2.41953000	-0.27206200
C	-1.53888100	1.96382400	-2.01943500
C	-3.53524800	3.59137000	-0.93464100
H	-3.64902900	2.15014900	0.66021100
C	-1.91957400	3.13197700	-2.67725300
H	-0.76277900	1.33282800	-2.44610200
C	-2.91621100	3.94796400	-2.13366000
H	-4.30927300	4.22417500	-0.51069800
H	-1.43731000	3.40685600	-3.61065700
H	-3.20795900	4.86085500	-2.64451700
C	3.79123000	0.42255300	0.00702400
C	4.99938400	-0.32390000	0.00825900
C	6.28362500	0.27085400	0.00442200
C	6.30751600	1.64179300	-0.00080400
C	5.09581700	2.41303000	-0.00250100

C	3.84662300	1.84156300	0.00115500
H	7.17759800	-0.34203400	0.00569000
H	7.25820400	2.16409300	-0.00386000
H	5.17806900	3.49560500	-0.00691000
H	2.93739000	2.43254500	-0.00062000
O	3.22970400	-1.69710800	0.01607900
N	2.74266600	-0.40328200	0.01198300
N	4.66989900	-1.63502600	0.01375600
O	5.29342400	-2.66939700	0.01683900

f

SCF done: -913.179089

Au	-1.91807600	-0.02660300	-0.00418800
P	0.36926300	0.00254900	0.00057500
C	0.98837600	1.47164700	-0.89843700
C	2.03754900	1.36291900	-1.82274400
C	0.41323700	2.72638300	-0.63409700
C	2.50484300	2.50477700	-2.47660300
H	2.48539500	0.39805500	-2.03640000
C	0.88842800	3.86076800	-1.28857300
H	-0.39963900	2.81700900	0.08233200
C	1.93284100	3.75009100	-2.21180100
H	3.31503100	2.41757400	-3.19417300
H	0.44177600	4.82874500	-1.08244400
H	2.29735200	4.63479600	-2.72532300
C	1.01299500	0.06379200	1.71250300
C	0.43562300	-0.76271400	2.69116900
C	2.09455300	0.89317200	2.04554800
C	0.93927800	-0.75890200	3.99029900
H	-0.40233100	-1.40820900	2.43907900
C	2.59053700	0.89282700	3.35083000
H	2.54472600	1.53804700	1.29776100
C	2.01533500	0.07042200	4.32111500
H	0.49067400	-1.39852200	4.74447300
H	3.42557800	1.53792300	3.60707300
H	2.40245400	0.07616700	5.33570300
C	1.03494500	-1.49632400	-0.81008500
C	2.15555300	-2.15758300	-0.28484300
C	0.43287700	-1.96411500	-1.99043100
C	2.66634100	-3.27961100	-0.93984200
H	2.62411200	-1.80673800	0.62897600
C	0.95201900	-3.08288700	-2.63909600
H	-0.43629000	-1.45712700	-2.40233600
C	2.06710700	-3.74196000	-2.11304200

H	3.53112500	-3.79238100	-0.52952700
H	0.48351100	-3.44309400	-3.55001300
H	2.46563600	-4.61773900	-2.61652000
O	-4.14298300	-0.07999500	-0.03877700
H	-4.55017100	-0.53458800	0.71956300
H	-4.56063100	0.79722400	-0.09704800

$Au^{+}PPh_3$

SCF done: -836.722393

Au	0.00734100	0.00777900	2.06829400
P	-0.00076100	-0.00097800	-0.23212200
C	-0.71181700	1.56744900	-0.82153800
C	-1.93103100	2.01447100	-0.28107100
C	-0.06775500	2.31000000	-1.82434100
C	-2.50219000	3.19472700	-0.74905100
H	-2.42775800	1.44592100	0.50109900
C	-0.64962900	3.49250300	-2.28385300
H	0.87436600	1.97312700	-2.24349200
C	-1.86050500	3.93487400	-1.74815400
H	-3.44204100	3.54127400	-0.33056300
H	-0.15269200	4.06756000	-3.05910400
H	-2.30475800	4.85916800	-2.10511600
C	-1.00911000	-1.40185300	-0.80968900
C	-1.97152500	-1.21868900	-1.81569900
C	-0.79244900	-2.67823700	-0.26005700
C	-2.70798900	-2.31411300	-2.26920200
H	-2.14628300	-0.23633400	-2.24139500
C	-1.53232700	-3.76340600	-0.72201900
H	-0.05375600	-2.82170500	0.52455000
C	-2.49132100	-3.58109400	-1.72446600
H	-3.45256700	-2.17366400	-3.04672600
H	-1.36718600	-4.74827700	-0.29622800
H	-3.07192700	-4.42831300	-2.07691700
C	1.71185300	-0.17529500	-0.82300900
C	2.02729600	-1.10469100	-1.82724800
C	2.71287000	0.65304700	-0.28436400
C	3.34083400	-1.19651200	-2.28999800
H	1.26117300	-1.74908200	-2.24509400
C	4.01919100	0.55312000	-0.75552200
H	2.47335200	1.36821500	0.49861100
C	4.33362300	-0.37294600	-1.75618700
H	3.58587700	-1.91489000	-3.06623900
H	4.79247700	1.19067900	-0.33827400
H	5.35505900	-0.45370500	-2.11569200

1

SCF done: -1748.075854

C	1.42817000	5.46809700	-0.69774800
C	0.63816900	4.34620700	-0.92251900
C	0.09386600	3.63456000	0.17109000
C	0.36510200	4.06837600	1.48864700
C	1.15963900	5.18881600	1.70153200
C	1.68974700	5.88806400	0.61119100
H	1.84088000	6.01741800	-1.53839700
H	0.42675900	4.00816600	-1.93218100
H	-0.05517900	3.51797200	2.32424800
H	1.36642900	5.52186200	2.71393000
H	2.30858100	6.76417900	0.78242200
C	-0.74968300	2.51536300	-0.05859700
C	-1.48166300	1.51329600	-0.25853600
S	-3.00630000	-0.52785900	-1.12173500
O	-3.28128600	-0.40226400	-2.56235200
O	-1.85595500	-1.31463800	-0.61723100
C	-4.49081500	-1.04307000	-0.27793000
C	-5.66748300	-1.19003900	-1.01486200
C	-4.43812900	-1.34604600	1.08719100
C	-6.81643900	-1.63260300	-0.36035400
H	-5.67592900	-0.97000300	-2.07691800
C	-5.59703600	-1.78416700	1.71759900
H	-3.50906100	-1.24638300	1.63856000
C	-6.80208200	-1.93606400	1.00817700
H	-7.73720300	-1.74738800	-0.92541200
H	-5.56886100	-2.01940700	2.77820500
C	-8.04138200	-2.44255000	1.70334400
H	-8.94841500	-2.17703000	1.15263800
H	-8.01640800	-3.53664800	1.78976500
H	-8.12434300	-2.03953400	2.71802200
N	-2.76013900	1.08314200	-0.46134200
C	-3.75622800	2.10890700	-0.89552700
H	-3.45582000	2.53204200	-1.86076100
H	-4.70052000	1.57327100	-1.03926700
Au	0.34348200	0.26884200	-0.09796700
P	2.29080600	-1.02249800	0.04871100
C	3.19662200	-0.70190500	1.61693500
C	4.59507600	-0.60329500	1.65093600
C	2.46246800	-0.58452800	2.80831500
C	5.24920400	-0.39256100	2.86693300
H	5.17275800	-0.68534100	0.73571700
C	3.12194900	-0.38002700	4.01916600

H	1.37744600	-0.65373500	2.78866000
C	4.51599700	-0.28217500	4.04936600
H	6.33231800	-0.31446500	2.88693500
H	2.54862500	-0.29281700	4.93756800
H	5.02823600	-0.11744800	4.99286800
C	1.95962300	-2.82686000	-0.00994900
C	0.88795400	-3.30520400	-0.78061600
C	2.78587200	-3.73222600	0.67529300
C	0.65504000	-4.67781500	-0.86999000
H	0.22669000	-2.61480500	-1.29525700
C	2.54370800	-5.10308400	0.58162900
H	3.60922000	-3.37176200	1.28410200
C	1.48100600	-5.57653800	-0.19133300
H	-0.17756300	-5.04205900	-1.46449000
H	3.18368500	-5.79941000	1.11566000
H	1.29348000	-6.64428100	-0.25936700
C	3.45369900	-0.63579300	-1.32217900
C	4.15038100	-1.64607500	-2.00050300
C	3.65480600	0.70778000	-1.67972300
C	5.03993100	-1.31216600	-3.02413800
H	3.99828500	-2.68792300	-1.73808000
C	4.54900100	1.03387500	-2.69781700
H	3.11324600	1.49673200	-1.16343500
C	5.24085500	0.02406700	-3.37268100
H	5.57273700	-2.09940000	-3.54937100
H	4.70114100	2.07449400	-2.96928700
H	5.93141700	0.27939500	-4.17116700
C	-3.92171600	3.17575900	0.14599600
H	-4.23806100	2.83481300	1.13083300
C	-3.73430700	4.47420900	-0.09519000
H	-3.42606500	4.83599500	-1.07378500
H	-3.90151600	5.22587600	0.67068900

TS1-2

SCF done: -1748.074743

C	1.74804800	5.59295400	-1.03557400
C	0.79949200	4.57533800	-1.05966200
C	0.61214600	3.76059300	0.07765200
C	1.39449600	3.98189900	1.23026200
C	2.33894200	5.00319100	1.24347600
C	2.51667400	5.80768600	0.11306200
H	1.88849000	6.22017900	-1.91063500
H	0.19737300	4.39917200	-1.94549000
H	1.24652600	3.35239900	2.10204100

H	2.93685200	5.17479800	2.13334700
H	3.25531400	6.60387100	0.12754900
C	-0.39223700	2.74213200	0.05990100
C	-1.33673700	1.95379900	-0.14420900
S	-3.08750400	0.36687100	-1.38945500
O	-3.75466500	1.10650900	-2.47207100
O	-1.85900100	-0.42208400	-1.62041500
C	-4.28913300	-0.62514300	-0.52227500
C	-5.63732700	-0.50223400	-0.86357400
C	-3.85833500	-1.54084300	0.44444500
C	-6.57064000	-1.30269200	-0.20568900
H	-5.94242500	0.19778100	-1.63388600
C	-4.80784800	-2.32653300	1.08739800
H	-2.80347800	-1.64137500	0.67799100
C	-6.17567100	-2.22261000	0.77534600
H	-7.62195300	-1.21257200	-0.46409700
H	-4.48552400	-3.03899300	1.84222800
C	-7.18646500	-3.10440300	1.46557800
H	-8.20374500	-2.72173900	1.34430000
H	-7.16313700	-4.11987200	1.04953600
H	-6.97684500	-3.18926100	2.53718000
N	-2.62384400	1.54074700	-0.14215700
C	-3.66411200	2.56978500	0.18101200
H	-3.65081300	3.36147400	-0.57603900
H	-4.62573000	2.04989900	0.11307300
Au	0.43049600	0.48975400	-0.01410500
P	2.00424300	-1.23392000	0.04509200
C	3.05048800	-1.16567400	1.55393300
C	4.41470900	-1.48972200	1.50801800
C	2.46106500	-0.81007500	2.77792600
C	5.17668600	-1.46136500	2.67781900
H	4.88329300	-1.75678700	0.56602800
C	3.22670100	-0.78885700	3.94280000
H	1.40663100	-0.54868500	2.81966700
C	4.58531600	-1.11284700	3.89341200
H	6.23311800	-1.70976400	2.63612100
H	2.76494700	-0.51416900	4.88672400
H	5.18203100	-1.08952200	4.80062300
C	1.21092200	-2.88951500	0.01117800
C	0.09597800	-3.09116700	-0.81990700
C	1.72030500	-3.95458900	0.76946100
C	-0.49276500	-4.35354900	-0.89488200
H	-0.31662000	-2.27098800	-1.40004500
C	1.12049500	-5.21285400	0.69090600

H	2.57607600	-3.80578700	1.42014100
C	0.01735700	-5.41385400	-0.14105500
H	-1.35236700	-4.50574000	-1.54108300
H	1.51714900	-6.03424500	1.28046100
H	-0.44565700	-6.39469900	-0.20029900
C	3.13539200	-1.14140700	-1.39854200
C	3.54716700	-2.29595200	-2.08008600
C	3.60937000	0.11596500	-1.80774900
C	4.42736900	-2.18956000	-3.15891500
H	3.18034800	-3.27170500	-1.77809800
C	4.49132000	0.21354000	-2.88249600
H	3.28939200	1.01628800	-1.28875600
C	4.89934900	-0.93877500	-3.56015700
H	4.73980500	-3.08584500	-3.68663400
H	4.85377400	1.18828500	-3.19544400
H	5.58042500	-0.86018400	-4.40249200
C	-3.47101000	3.12147600	1.56267500
H	-3.50065000	2.39596600	2.37439400
C	-3.30803900	4.42003300	1.81828500
H	-3.28266900	5.16195400	1.02306400
H	-3.21330000	4.79253800	2.83405200

2

SCF done: -1748.090121

C	0.86048200	5.13990400	0.28855800
C	0.55535100	3.78052900	0.32989700
C	1.55034900	2.83213200	0.62499800
C	2.86128600	3.28471600	0.87990600
C	3.16252200	4.64136900	0.83977200
C	2.16232400	5.57384400	0.54369000
H	0.08065200	5.85968100	0.05834400
H	-0.45834400	3.44507300	0.13109300
H	3.64146900	2.56390700	1.11126500
H	4.17625900	4.97528500	1.04101500
H	2.39901700	6.63335100	0.51377100
C	1.23447600	1.40188000	0.66614400
C	1.97611700	0.37935000	0.90483000
S	3.18041700	-1.61529300	-0.49482900
O	3.06601900	-3.02945400	-0.10969700
O	2.38285600	-0.97181700	-1.54414200
C	4.88707600	-1.14551100	-0.63111200
C	5.87140200	-2.00075000	-0.12714000
C	5.21001300	0.06234800	-1.26042400
C	7.20603600	-1.62251100	-0.24871300

H	5.59853300	-2.94615000	0.32868500
C	6.55064200	0.41658700	-1.36601600
H	4.43108200	0.69718800	-1.66925100
C	7.56808200	-0.41488200	-0.86590300
H	7.98030900	-2.28085500	0.13506300
H	6.81550900	1.35014400	-1.85485900
C	9.01796500	-0.03124500	-1.02182300
H	9.64414600	-0.50523000	-0.26034200
H	9.39596500	-0.34864000	-2.00246000
H	9.15507300	1.05269800	-0.95665900
N	2.62812300	-0.70570900	1.05822100
C	2.77847800	-1.41633300	2.37568000
H	3.44925200	-2.25798000	2.20083100
H	3.27059700	-0.71021400	3.05188400
Au	-0.68772900	0.57153100	0.26568800
P	-2.83190900	-0.25446000	-0.23051800
C	-3.90936400	-0.35157800	1.25574300
C	-4.85980200	-1.37257000	1.40691300
C	-3.78894600	0.63656900	2.24626900
C	-5.68162000	-1.39815700	2.53524800
H	-4.95569900	-2.14724400	0.65248000
C	-4.61585200	0.60777900	3.36873600
H	-3.04889500	1.42610600	2.14124900
C	-5.56194300	-0.41002000	3.51454000
H	-6.41338500	-2.19274700	2.64839400
H	-4.51744300	1.37546500	4.13071200
H	-6.20156400	-0.43494100	4.39203600
C	-3.69350000	0.81943800	-1.44902300
C	-2.96659200	1.29174100	-2.55489800
C	-5.04536500	1.16387900	-1.30837000
C	-3.58974900	2.09254100	-3.51064400
H	-1.91709700	1.03141400	-2.66995900
C	-5.66169400	1.97134500	-2.26708200
H	-5.61545800	0.81005500	-0.45533500
C	-4.93732400	2.43477700	-3.36638400
H	-3.02283200	2.45275900	-4.36414300
H	-6.70828100	2.23766400	-2.15149900
H	-5.41992800	3.06379500	-4.10878000
C	-2.76971000	-1.93871000	-0.96208400
C	-3.66610600	-2.33371300	-1.96669400
C	-1.81065800	-2.84877400	-0.48885300
C	-3.60455000	-3.62840000	-2.48532900
H	-4.40279700	-1.63438700	-2.34988300
C	-1.75651000	-4.14082300	-1.00999000

H	-1.10428600	-2.54847300	0.28121900
C	-2.65229300	-4.53132400	-2.00863000
H	-4.29840500	-3.92782600	-3.26540400
H	-1.00875000	-4.83816200	-0.64389700
H	-2.60365200	-5.53586400	-2.41881300
C	1.45905900	-1.88397800	2.92218200
H	0.76946400	-1.10503700	3.24398400
C	1.13267500	-3.17116500	3.04699100
H	1.80511000	-3.96679400	2.73513900
H	0.18251100	-3.47496300	3.47707400

3

SCF done: -2238.929610

C	-0.48555800	4.22841900	-2.61283900
C	-0.02154000	3.00484600	-2.13363800
C	-0.92221500	1.96556200	-1.84038500
C	-2.30102000	2.18535100	-2.03312700
C	-2.76212700	3.41175100	-2.50112000
C	-1.85443800	4.43537000	-2.79471700
H	0.22157200	5.02044700	-2.84116800
H	1.04363000	2.84462900	-1.99210500
H	-3.00781200	1.39702400	-1.79488900
H	-3.82859900	3.57503300	-2.62774900
H	-2.21608500	5.39133800	-3.16227500
C	-0.43729500	0.67684200	-1.34585400
C	-1.00363600	-0.46282600	-1.17163900
S	-1.84432800	-2.14493000	0.78015600
O	-0.81262500	-3.14450700	1.11127600
O	-1.95252300	-0.84981700	1.46202500
C	-3.41991900	-2.93884300	0.59662900
C	-3.48129700	-4.33410300	0.67625800
C	-4.56252900	-2.15257900	0.40773500
C	-4.72354200	-4.95097200	0.55072100
H	-2.58140800	-4.91454400	0.84775500
C	-5.78789100	-2.79848000	0.28250000
H	-4.50243500	-1.06848900	0.36430600
C	-5.89206900	-4.19882300	0.35192900
H	-4.78717500	-6.03358100	0.61563200
H	-6.68370200	-2.20187200	0.13294600
C	-7.23709100	-4.87193500	0.24304900
H	-7.78521800	-4.79292400	1.19079100
H	-7.85697400	-4.40235300	-0.52783200
H	-7.13877500	-5.93522800	0.00646400
N	-1.41874100	-1.65405900	-0.97131200

C	-1.29386500	-2.74737400	-1.99359900
H	-0.26348900	-2.70582600	-2.36408700
H	-1.42347900	-3.69463900	-1.46638900
Au	1.53293900	0.31332000	-0.60553200
P	3.67908200	-0.08746000	0.26253800
C	3.59926600	-0.94013500	1.88794500
C	4.57171000	-0.71788000	2.87552200
C	2.55318800	-1.84409800	2.13363000
C	4.49829800	-1.39954900	4.09121500
H	5.37776700	-0.01153600	2.70186900
C	2.48718300	-2.52252500	3.35032800
H	1.78472900	-2.01969500	1.38578300
C	3.45848100	-2.30122100	4.32942300
H	5.25181000	-1.22145000	4.85295000
H	1.67043500	-3.21499500	3.53086400
H	3.40250100	-2.82573800	5.27899300
C	4.68182200	-1.14435900	-0.85965600
C	4.62382100	-0.90043500	-2.24197600
C	5.50387100	-2.16984400	-0.37058000
C	5.38701000	-1.66737700	-3.12101300
H	3.98261700	-0.11285700	-2.63040300
C	6.26100800	-2.93856600	-1.25729500
H	5.55091100	-2.37390600	0.69437200
C	6.20486600	-2.68860400	-2.62943100
H	5.33870000	-1.47196200	-4.18831700
H	6.89308400	-3.73379300	-0.87290100
H	6.79411900	-3.28983600	-3.31576600
C	4.63107400	1.46346600	0.52151300
C	5.99731700	1.55084900	0.21733600
C	3.96556700	2.57886000	1.05669100
C	6.68824300	2.74203000	0.45011800
H	6.52065900	0.69846400	-0.20415200
C	4.66239800	3.76335100	1.29054700
H	2.90550700	2.52080900	1.29198000
C	6.02394600	3.84647600	0.98594200
H	7.74552800	2.80491100	0.20963500
H	4.14212400	4.62185900	1.70524200
H	6.56422700	4.77192900	1.16301800
C	-2.29315000	-2.59263300	-3.10387400
C	-3.20961600	-3.51223200	-3.40420100
H	-2.21683500	-1.67665400	-3.68841900
H	-3.89529700	-3.37845700	-4.23563300
H	-3.30448900	-4.43614800	-2.83812800
C	-4.45996300	2.13025600	1.40872600

C	-4.93033000	3.43271900	1.06996500
C	-4.80954900	4.55091500	1.93205400
C	-4.20137400	4.31671100	3.13703000
C	-3.70791700	3.01174300	3.49527200
C	-3.81897300	1.92544900	2.66882300
H	-5.18646500	5.52268600	1.63407300
H	-4.08119500	5.13011900	3.84539500
H	-3.22735600	2.89513000	4.46215400
H	-3.43225300	0.94687500	2.92940400
O	-5.32958900	1.97005400	-0.56004100
N	-4.70033600	1.26036200	0.43590300
N	-5.46458100	3.35347900	-0.15982900
O	-5.97493800	4.14699200	-0.93650600

Ts3-4

SCF done:-2238.907295

C	0.59646200	-4.92285000	0.72803300
C	0.45124400	-3.53656200	0.75990400
C	0.82953100	-2.75487300	-0.34605500
C	1.31893000	-3.39793700	-1.49657400
C	1.44417100	-4.78762800	-1.52933400
C	1.09064400	-5.55376000	-0.41653300
H	0.31148700	-5.51230100	1.59505800
H	0.04992800	-3.04930200	1.64431100
H	1.58195100	-2.80894200	-2.36972600
H	1.81407800	-5.27085100	-2.42938100
H	1.18739500	-6.63517100	-0.44531600
C	0.65194200	-1.29042900	-0.31187900
C	1.56757000	-0.35039200	-0.38782300
S	1.86722900	1.94394200	0.93361600
O	0.70229700	2.84980600	0.93963800
O	2.09858000	0.95141100	1.99803800
C	3.33621800	2.91806700	0.66133200
C	3.20448200	4.27868900	0.37885000
C	4.59276200	2.31594500	0.78246900
C	4.35789500	5.04029700	0.19666900
H	2.22064600	4.73102900	0.32178300
C	5.72860600	3.09486800	0.59417800
H	4.67677500	1.26387100	1.03108600
C	5.63264400	4.46517300	0.29418200
H	4.26412800	6.10173100	-0.01555300
H	6.70958400	2.63705500	0.69045100
C	6.87666600	5.28968600	0.07425600
H	7.65679100	5.03614300	0.79979800

H	7.29251400	5.10723200	-0.92516500
H	6.66911200	6.36044700	0.15503500
N	1.71809500	0.96222800	-0.53210100
C	1.47906900	1.65057000	-1.83410700
H	0.62032000	1.14382700	-2.29069700
H	1.16614300	2.67267400	-1.60559100
Au	-1.30178100	-0.53544500	-0.13087800
P	-3.50551300	0.29033500	0.05969700
C	-3.72341800	1.36732300	1.53316800
C	-4.92686700	1.39181100	2.25480100
C	-2.65666000	2.19119000	1.92873600
C	-5.06060500	2.23684800	3.35772000
H	-5.75367500	0.75099300	1.96470300
C	-2.80065700	3.03564300	3.02961300
H	-1.71447300	2.17789800	1.38773900
C	-4.00007400	3.05867800	3.74527800
H	-5.99292000	2.24906100	3.91496500
H	-1.96991400	3.66753900	3.32965000
H	-4.10688200	3.71188400	4.60670000
C	-3.97293400	1.29498700	-1.41065200
C	-3.64131300	0.80835200	-2.68629400
C	-4.65284000	2.51512300	-1.29094300
C	-3.99465300	1.53014000	-3.82536400
H	-3.11029600	-0.13518800	-2.78791900
C	-4.99859800	3.23675300	-2.43598300
H	-4.90908000	2.90452000	-0.31076900
C	-4.67195400	2.74660300	-3.70123900
H	-3.73833800	1.14560500	-4.80842500
H	-5.52354800	4.18239400	-2.33578100
H	-4.94218600	3.31070400	-4.58931400
C	-4.76192000	-1.04684600	0.18794700
C	-6.01837100	-0.94131500	-0.42698500
C	-4.45404900	-2.18972900	0.94326100
C	-6.95436600	-1.96741700	-0.28269200
H	-6.26527200	-0.06687900	-1.02102900
C	-5.39452900	-3.20884800	1.08761100
H	-3.47952900	-2.28243500	1.41599800
C	-6.64489800	-3.09930000	0.47359500
H	-7.92416100	-1.88127700	-0.76420700
H	-5.14904900	-4.09027400	1.67292100
H	-7.37441900	-3.89683800	0.58116500
C	2.67515100	1.63413400	-2.74524200
C	3.20953500	2.72994700	-3.28373800
H	3.08275300	0.65283400	-2.98360500

H	4.04894000	2.67478900	-3.97082900
H	2.82354500	3.72372500	-3.06727400
C	3.84082500	-1.91657700	0.62734900
C	4.74225600	-2.85008700	0.04839500
C	5.46494600	-3.80531500	0.80245500
C	5.24853800	-3.78431800	2.15615000
C	4.34378100	-2.84486900	2.75833400
C	3.63701000	-1.91602200	2.03431300
H	6.14406200	-4.49633100	0.31658700
H	5.77289800	-4.48688100	2.79538200
H	4.21972100	-2.87382200	3.83667600
H	2.96587100	-1.19618200	2.48707800
O	3.84501300	-1.53808800	-1.52765500
N	3.32628400	-1.12208200	-0.31847800
N	4.74647300	-2.64393700	-1.28558100
O	5.32084000	-3.14427900	-2.22630700

4

SCF done: -2238.923159

C	0.34433800	-5.09069600	0.55229300
C	0.24562700	-3.70080100	0.58751500
C	0.86325700	-2.90966100	-0.40011400
C	1.54746600	-3.56037300	-1.44553800
C	1.61848200	-4.95293600	-1.49353000
C	1.02717400	-5.72316200	-0.49032900
H	-0.12681500	-5.68247700	1.33223800
H	-0.30707800	-3.21510000	1.38770600
H	1.99395900	-2.97190600	-2.24252500
H	2.13184400	-5.43612900	-2.32039900
H	1.08426600	-6.80707600	-0.52817300
C	0.74927300	-1.43334800	-0.35667800
C	1.84757800	-0.64129000	-0.42933500
S	1.87191000	1.65940300	0.89904100
O	0.51680300	2.22158700	1.09704900
O	2.47593600	0.74611100	1.89515900
C	2.98691800	3.02413100	0.58555700
C	2.46802600	4.30609100	0.40400500
C	4.36620500	2.79217200	0.58004100
C	3.35060000	5.36680900	0.19583200
H	1.39624500	4.46751800	0.44043500
C	5.22795900	3.86240000	0.37154200
H	4.75258200	1.79335000	0.75278200
C	4.73668400	5.16527800	0.17235200
H	2.95291900	6.36845900	0.05780500

H	6.30135000	3.69092800	0.37097700
C	5.68852700	6.31221000	-0.06373400
H	6.49240900	6.32068600	0.68082600
H	6.16327400	6.23081400	-1.04976300
H	5.17489800	7.27667100	-0.02025100
N	1.92908200	0.76444400	-0.59071900
C	1.23455100	1.41827200	-1.73648100
H	0.14816800	1.39913500	-1.57876800
H	1.55388800	2.46463700	-1.73743000
Au	-1.15118600	-0.63174100	-0.16239000
P	-3.34428400	0.24269800	0.09445100
C	-3.65769400	0.82277400	1.81286900
C	-4.89044500	0.62167100	2.45127500
C	-2.62238700	1.49144900	2.48922900
C	-5.08674000	1.08883800	3.75232800
H	-5.69310000	0.09904700	1.94076300
C	-2.83057200	1.96009500	3.78654700
H	-1.65983700	1.64994600	2.00964900
C	-4.05954100	1.75831400	4.41991200
H	-6.04248300	0.92691200	4.24257700
H	-2.02752600	2.47753700	4.30357700
H	-4.21510000	2.11893100	5.43283400
C	-3.64561400	1.68806600	-1.00826300
C	-3.22737200	1.60324200	-2.34671200
C	-4.28572800	2.85029700	-0.55677000
C	-3.45506400	2.66466800	-3.22144500
H	-2.72927800	0.70521200	-2.70474000
C	-4.50639000	3.91298600	-1.43644700
H	-4.60825900	2.92957000	0.47652700
C	-4.09341500	3.82197600	-2.76641000
H	-3.13219300	2.58985200	-4.25596700
H	-5.00188300	4.81121500	-1.07913000
H	-4.26679700	4.65021100	-3.44757500
C	-4.66620700	-0.97243900	-0.31102100
C	-5.90518200	-0.56473000	-0.82962700
C	-4.42962500	-2.33668300	-0.08008000
C	-6.89411000	-1.51079700	-1.10376900
H	-6.09602100	0.48573300	-1.02730900
C	-5.42265800	-3.27756400	-0.35286000
H	-3.46753500	-2.66350400	0.30602300
C	-6.65518900	-2.86586400	-0.86483700
H	-7.84940000	-1.18814100	-1.50774300
H	-5.23052800	-4.33151000	-0.17351600
H	-7.42554000	-3.59990300	-1.08317900

C	1.59259900	0.78017500	-3.05076700
C	2.28677900	1.39618900	-4.00789700
H	1.21854300	-0.22853300	-3.21741900
H	2.49500400	0.91542800	-4.95967900
H	2.66616500	2.40807300	-3.88207100
C	3.77522700	-1.90331200	0.68200300
C	5.03601900	-2.35054700	0.21177300
C	5.94523300	-3.08875000	1.00532400
C	5.53304400	-3.35662400	2.28564000
C	4.26449000	-2.89878600	2.77561400
C	3.37576200	-2.17990000	2.01043700
H	6.89927400	-3.40883500	0.60330600
H	6.17815400	-3.91938700	2.95182500
H	4.00064000	-3.12643400	3.80374400
H	2.43400800	-1.80762200	2.38919400
O	3.99676500	-1.21798600	-1.43089600
N	3.15637400	-1.23747500	-0.30150600
N	5.18305800	-1.93066800	-1.06873800
O	6.06703000	-2.02343600	-1.88502600

TS4-5

SCF done: -2238.918353

C	0.37128100	-5.12003500	0.13608500
C	0.22984900	-3.74171900	0.26340300
C	0.97271400	-2.86001000	-0.55180400
C	1.82394500	-3.41500200	-1.53365800
C	1.92860400	-4.79611500	-1.68597100
C	1.21499900	-5.65278600	-0.84408600
H	-0.19135100	-5.78222500	0.78785300
H	-0.44403900	-3.32931000	1.00918600
H	2.36646900	-2.75721800	-2.20507700
H	2.56460700	-5.20462600	-2.46622000
H	1.30256300	-6.72902800	-0.96093500
C	0.80910500	-1.40872400	-0.41450800
C	1.90122900	-0.55887600	-0.41505700
S	1.74253600	1.80388300	0.81364000
O	0.38439200	2.39074000	0.89078000
O	2.26110700	0.93493800	1.89254900
C	2.89408200	3.13920900	0.50840700
C	2.40607300	4.42363600	0.26586600
C	4.26724300	2.88132000	0.56136600
C	3.31552700	5.46111900	0.05986200
H	1.33672700	4.60296600	0.24885600
C	5.15631000	3.92995200	0.35408900

H	4.62777900	1.87898700	0.76650400
C	4.69794200	5.23501500	0.10205600
H	2.94244500	6.46364800	-0.13135500
H	6.22543100	3.73800400	0.39211800
C	5.67651000	6.36780500	-0.08874300
H	6.56618900	6.04063800	-0.63679300
H	5.22507200	7.20195300	-0.63397600
H	6.01655300	6.75425600	0.88091500
N	1.86386200	0.84338000	-0.62453200
C	1.18655700	1.41476500	-1.82129200
H	0.09710400	1.40238000	-1.68737900
H	1.50012600	2.46141700	-1.88308800
Au	-1.10103200	-0.64834500	-0.17463200
P	-3.30051100	0.21444800	0.11158000
C	-3.60244900	0.76669900	1.84070500
C	-4.80710200	0.49566300	2.50585900
C	-2.58593200	1.48179200	2.49839900
C	-4.99402500	0.93776900	3.81712400
H	-5.59546900	-0.05966500	2.00798200
C	-2.78564600	1.92478600	3.80587500
H	-1.64705300	1.69914900	1.99528800
C	-3.98599500	1.65179500	4.46722400
H	-5.92792200	0.72219700	4.32831100
H	-1.99839100	2.47910000	4.30873500
H	-4.13426700	1.99315900	5.48785100
C	-3.60433400	1.67435100	-0.96943000
C	-3.19850900	1.60523300	-2.31258400
C	-4.23644900	2.83230800	-0.49656400
C	-3.42990600	2.67896400	-3.17109900
H	-2.70783300	0.70987500	-2.68733800
C	-4.46092200	3.90719600	-1.36023300
H	-4.54909400	2.89902100	0.54060300
C	-4.05980000	3.83237800	-2.69482300
H	-3.11667500	2.61677900	-4.20938100
H	-4.94992700	4.80234000	-0.98673200
H	-4.23594300	4.67027600	-3.36329400
C	-4.62186000	-0.99634200	-0.30652100
C	-5.86390500	-0.57960700	-0.81047400
C	-4.38504300	-2.36418500	-0.09893500
C	-6.85448200	-1.52073400	-1.09513400
H	-6.05595000	0.47420200	-0.98782000
C	-5.37973400	-3.30029900	-0.38174100
H	-3.42167500	-2.69732000	0.27835800
C	-6.61469600	-2.87971600	-0.88046300

H	-7.81198900	-1.19106400	-1.48793100
H	-5.18764800	-4.35713500	-0.22024100
H	-7.38647700	-3.60977000	-1.10681200
C	1.57397700	0.69697300	-3.08611200
C	2.32931500	1.23757400	-4.04248600
H	1.17209700	-0.30647000	-3.21848700
H	2.56107900	0.70003400	-4.95771200
H	2.73951500	2.24108100	-3.95160900
C	3.64382000	-1.85478400	0.84775200
C	4.85371400	-2.46659800	0.45560600
C	5.60938400	-3.31396000	1.28952700
C	5.10309500	-3.53631100	2.54863000
C	3.88685900	-2.91794200	2.96507200
C	3.14749400	-2.08352800	2.15075500
H	6.53464900	-3.75152400	0.93269400
H	5.63362600	-4.18136700	3.24047600
H	3.52983300	-3.10722900	3.97301700
H	2.24567000	-1.58578600	2.48175700
O	4.22929800	-1.25339000	-1.32814300
N	3.16146200	-1.02825500	-0.10191900
N	5.17140300	-2.07485700	-0.82287800
O	6.15429300	-2.34695900	-1.48277700

5

SCF done: -2238.963844

C	0.08121400	-4.48278800	-2.71229500
C	-0.17076600	-3.32882100	-1.99377000
C	0.89667900	-2.45556400	-1.61420900
C	2.23159500	-2.80990800	-1.99016100
C	2.47157000	-3.96894200	-2.70815100
C	1.40158100	-4.80107600	-3.07058200
H	-0.73300300	-5.14004100	-2.99994600
H	-1.18452900	-3.06448600	-1.70879200
H	3.06113800	-2.17922000	-1.68610300
H	3.48584900	-4.23413600	-2.98891000
H	1.59717000	-5.70786600	-3.63653500
C	0.61243300	-1.26006600	-0.91486000
C	1.71106300	-0.35698400	-0.46508400
S	1.93955100	2.31841600	-0.00425200
O	1.46917100	3.42523300	-0.85465200
O	1.27640700	1.97945900	1.27047900
C	3.69337400	2.49517300	0.26799200
C	4.45689200	3.16374300	-0.69203900
C	4.26000600	2.01636500	1.45092900

C	5.81995900	3.33044300	-0.46294500
H	3.99245100	3.54304800	-1.59511100
C	5.62377300	2.20059200	1.65976400
H	3.64224200	1.51478700	2.18594800
C	6.42478700	2.85557200	0.71112000
H	6.42390900	3.84463900	-1.20577100
H	6.07539700	1.83083300	2.57645000
C	7.89676300	3.07106700	0.96295800
H	8.32413400	2.25909400	1.55961300
H	8.45811200	3.14093800	0.02620400
H	8.06325100	4.00532300	1.51518400
N	1.72072800	0.90738100	-1.04016400
C	1.34318200	1.12667100	-2.45622600
H	0.78482800	0.23375600	-2.76953200
H	0.65444500	1.97097900	-2.51339300
Au	-1.23977600	-0.54978800	-0.37350200
P	-3.33191400	0.38917900	0.28552900
C	-3.22751500	1.20964900	1.92487000
C	-4.32193000	1.22627000	2.80382500
C	-2.02998200	1.85001700	2.28519500
C	-4.21733900	1.88326600	4.03054700
H	-5.24807000	0.72666600	2.53683600
C	-1.93674900	2.50692200	3.51256000
H	-1.16729300	1.84213300	1.62429800
C	-3.02730500	2.52345500	4.38510000
H	-5.06543700	1.89231600	4.70906100
H	-1.00719300	2.99868200	3.78305800
H	-2.94971400	3.03125700	5.34223300
C	-3.88542200	1.65645000	-0.92742600
C	-3.80514100	1.35512500	-2.29754400
C	-4.38169100	2.90217000	-0.51919800
C	-4.22591500	2.28665300	-3.24501400
H	-3.41800900	0.39226500	-2.62296500
C	-4.79608800	3.83347100	-1.47416100
H	-4.44029800	3.14889900	0.53589500
C	-4.71975500	3.52807000	-2.83374100
H	-4.16393000	2.04673400	-4.30253800
H	-5.17606900	4.79856000	-1.15217300
H	-5.04088500	4.25614000	-3.57294200
C	-4.67711200	-0.86033700	0.38942300
C	-5.98842300	-0.56551700	-0.01185900
C	-4.38043500	-2.13095500	0.90908100
C	-6.98918900	-1.53189400	0.10968200
H	-6.22860000	0.41062700	-0.42148400

C	-5.38477800	-3.09021600	1.03202000
H	-3.36606700	-2.36798300	1.22148800
C	-6.68983100	-2.79182900	0.63095100
H	-8.00213300	-1.29823900	-0.20485300
H	-5.14893000	-4.07013300	1.43700600
H	-7.47060300	-3.54129900	0.72241700
C	2.53157600	1.33668100	-3.35529300
C	2.65082200	2.37395600	-4.18558600
H	3.30816900	0.57336900	-3.31034800
H	3.50597700	2.47855700	-4.84725700
H	1.89545000	3.15507100	-4.23473300
C	2.50613600	-1.74189900	1.32165800
C	3.66824000	-2.52364600	1.51953900
C	3.71381600	-3.54622000	2.47157400
C	2.59898300	-3.82024200	3.25327100
C	1.44435900	-3.04699100	3.09121300
C	1.40143000	-2.02205700	2.15142600
H	4.63300500	-4.10959900	2.57719700
H	2.63224900	-4.61934500	3.98628100
H	0.57220500	-3.23559800	3.71087100
H	0.52640200	-1.38487000	2.07296200
O	4.77639600	-1.71118300	-0.35528600
N	2.51826700	-0.62602500	0.49467400
N	4.87998400	-2.29563600	0.72996200
O	5.94309000	-2.70800700	1.18739600

TS5-6

SCF done: -2238.938167

C	0.43273400	3.62554900	3.14370000
C	0.14822900	2.77696500	2.08073800
C	1.13872700	1.92566100	1.53934500
C	2.42737000	1.95837300	2.12244700
C	2.70374200	2.79415100	3.20167000
C	1.71143600	3.63381400	3.71290500
H	-0.34329700	4.27408800	3.53949300
H	-0.84879800	2.76297000	1.64896500
H	3.21420300	1.31620500	1.73823700
H	3.69571000	2.79018800	3.64353800
H	1.92952200	4.28637500	4.55314400
C	0.80311800	1.01918400	0.42281100
C	1.90674700	0.08477700	-0.04327100
S	2.73905600	-2.45893000	-0.68228200
O	2.18303900	-3.70626500	-0.13115300
O	2.49767300	-2.02525100	-2.06153500

C	4.46184400	-2.36382800	-0.25175600
C	4.94689800	-3.21199000	0.75084400
C	5.30359700	-1.49344800	-0.95061600
C	6.30095500	-3.15931000	1.06914500
H	4.28396800	-3.91028900	1.24958200
C	6.65249700	-1.45994700	-0.61037500
H	4.90920200	-0.84982400	-1.72502500
C	7.17306600	-2.28686500	0.39820500
H	6.68982200	-3.81421500	1.84412000
H	7.30927800	-0.77621500	-1.14079100
C	8.64452100	-2.26081200	0.72947200
H	8.83558800	-2.61899900	1.74556600
H	9.20614000	-2.90703700	0.04227600
H	9.05795300	-1.25162000	0.63512400
N	1.91262900	-1.20999900	0.34633300
C	1.38873300	-1.67304700	1.66019100
H	2.17383100	-2.31109500	2.08317200
H	1.30898100	-0.79660900	2.30803600
Au	-1.17273000	0.35755100	0.16667200
P	-3.42094400	-0.32317000	-0.21913900
C	-3.55711900	-1.62082800	-1.51313000
C	-4.72954700	-1.77297400	-2.27108200
C	-2.46999100	-2.48020500	-1.73811700
C	-4.81119600	-2.77897800	-3.23438200
H	-5.57140900	-1.10442800	-2.11742700
C	-2.55799700	-3.48465600	-2.70255400
H	-1.55516400	-2.36235900	-1.16397200
C	-3.72786200	-3.63450300	-3.45035200
H	-5.71934900	-2.89040500	-3.81961300
H	-1.71077700	-4.14219800	-2.87351500
H	-3.79357100	-4.41298800	-4.20491300
C	-4.21429500	-0.98144700	1.30586800
C	-4.06022600	-0.26040300	2.50237000
C	-4.95104400	-2.17389900	1.30890300
C	-4.64537500	-0.72157500	3.68059900
H	-3.48385600	0.66171600	2.51237600
C	-5.52665000	-2.63671500	2.49509700
H	-5.07535600	-2.74333900	0.39354000
C	-5.37695800	-1.91296000	3.67894900
H	-4.52581000	-0.15567400	4.60004300
H	-6.09359200	-3.56311100	2.48983600
H	-5.82671500	-2.27542300	4.59872800
C	-4.48431700	1.06941800	-0.79069800
C	-5.75445200	1.31198800	-0.24980800

C	-4.00138700	1.89602800	-1.81916200
C	-6.52912400	2.37027200	-0.73204800
H	-6.14025200	0.68237100	0.54541100
C	-4.78165200	2.94512600	-2.30182200
H	-3.01984700	1.71035200	-2.24914900
C	-6.04605600	3.18580700	-1.75584400
H	-7.51157400	2.55301900	-0.30648200
H	-4.40468100	3.57493800	-3.10265200
H	-6.65161300	4.00649500	-2.12925900
C	0.08814000	-2.43301100	1.62526400
C	-0.89898300	-2.22110900	2.49846100
H	0.02808800	-3.23813300	0.89865500
H	-1.79549400	-2.83357800	2.50206900
H	-0.84611200	-1.43789900	3.25207100
C	2.54678000	1.78223500	-1.27029700
C	3.47525700	2.83881600	-1.47110400
C	3.03461700	4.14788200	-1.51216700
C	1.65460900	4.47114200	-1.47317100
C	0.71872600	3.46541400	-1.42554200
C	1.13807500	2.10472700	-1.32713700
H	3.78119000	4.93318700	-1.56341300
H	1.35628800	5.51215300	-1.53804400
H	-0.34220600	3.68941300	-1.48813000
H	0.50687500	1.36789800	-1.81353100
O	5.29793200	1.52062200	-1.98796700
N	2.86974200	0.54968300	-0.82898900
N	4.92406700	2.59695400	-1.53158300
O	5.65955100	3.50497000	-1.15024700

6

SCF done: -2238.968616

C	-0.35324500	5.09980000	1.12706600
C	-0.36706500	3.81167700	0.58619800
C	0.81235600	3.05752400	0.48901500
C	2.01283700	3.64415200	0.93287800
C	2.02623200	4.92666600	1.48249400
C	0.84156900	5.65935100	1.58230000
H	-1.27938600	5.66355000	1.19420800
H	-1.30370900	3.38525500	0.23656000
H	2.94535800	3.08968700	0.85245800
H	2.96342700	5.35331500	1.82843100
H	0.85075400	6.65828500	2.00805900
C	0.85141400	1.70507900	-0.18525000
C	1.69985200	0.60683700	0.29372500

S	2.55453200	-1.54596400	1.80463100
O	2.44548900	-1.65781200	3.26839400
O	1.78113000	-2.40594700	0.89881200
C	4.27548500	-1.51153200	1.35884400
C	5.20005900	-1.07950900	2.31559700
C	4.67640000	-2.01178200	0.11756000
C	6.55451600	-1.12894800	1.99993700
H	4.86890800	-0.73276500	3.28817300
C	6.03755900	-2.05785300	-0.16851800
H	3.94186300	-2.34268700	-0.60512000
C	6.99562200	-1.62009200	0.76025500
H	7.28266000	-0.79444400	2.73374800
H	6.35954400	-2.44483700	-1.13149400
C	8.46812800	-1.70909200	0.44560200
H	9.04816900	-0.98191700	1.02177800
H	8.85470800	-2.70663900	0.69212100
H	8.66153000	-1.54089500	-0.61854400
N	1.90991100	0.16243000	1.54329500
C	1.33359800	0.79652800	2.75652500
H	2.03075200	0.57610300	3.56801000
H	1.33169500	1.87420300	2.59715800
Au	-1.06586400	0.56630600	-0.10829100
P	-3.06886800	-0.67901200	-0.17953900
C	-2.74901000	-2.47982100	-0.33923600
C	-3.73699600	-3.34055400	-0.84663400
C	-1.50890500	-3.00182700	0.05999500
C	-3.48380300	-4.70830200	-0.94426200
H	-4.69470500	-2.94521800	-1.17192600
C	-1.26336700	-4.37200900	-0.04083200
H	-0.72503200	-2.35311000	0.43885800
C	-2.24860700	-5.22464200	-0.54179100
H	-4.24909000	-5.36954500	-1.34021200
H	-0.29712700	-4.76155000	0.26476200
H	-2.05394200	-6.28998300	-0.62575400
C	-4.06792100	-0.42466500	1.34424800
C	-4.44304900	0.88526100	1.69008200
C	-4.42967900	-1.49048800	2.17888100
C	-5.18185500	1.12022700	2.84820300
H	-4.16255100	1.72031100	1.05252400
C	-5.16210700	-1.24732700	3.34404200
H	-4.14474500	-2.50609000	1.92464100
C	-5.54007600	0.05338400	3.67887000
H	-5.47557600	2.13408600	3.10457400
H	-5.43858600	-2.07846900	3.98618200

H	-6.11209900	0.23793100	4.58352000
C	-4.15341800	-0.21075100	-1.59071300
C	-5.54876400	-0.14434700	-1.46180600
C	-3.56151100	0.04465200	-2.83827400
C	-6.33816300	0.17435300	-2.56910500
H	-6.02011100	-0.33541400	-0.50304600
C	-4.35480200	0.35709500	-3.94105700
H	-2.48095100	-0.00773100	-2.94748700
C	-5.74428500	0.42495500	-3.80692800
H	-7.41764600	0.22637100	-2.46116900
H	-3.88960300	0.55074300	-4.90330200
H	-6.36124400	0.67408300	-4.66537100
C	-0.04223800	0.30038700	3.12383100
C	-1.07301800	1.11532300	3.36219200
H	-0.14083200	-0.77325600	3.26857300
H	-2.03207000	0.73302300	3.69833400
H	-0.98964800	2.19375000	3.24555300
C	2.21828300	0.63094000	-1.81514700
C	2.93451500	0.52374600	-3.06193700
C	3.06285200	1.64327600	-3.83556000
C	2.44526600	2.90075200	-3.48662900
C	1.60479000	2.99284100	-2.42817800
C	1.22259700	1.74557600	-1.69329400
H	3.68934900	1.57763300	-4.71932400
H	2.66563500	3.76786400	-4.10064100
H	1.10700900	3.92308500	-2.17211500
H	0.34479200	1.39095300	-2.27921500
O	3.33655900	-1.73561200	-2.87036900
N	2.39167900	-0.05077400	-0.71919600
N	3.65307500	-0.69727300	-3.44644400
O	4.49573300	-0.60375400	-4.33586100

TS6-7

SCF done: -2729.790619

C	-0.35603000	-1.82232400	5.10890800
C	-0.36535300	-1.25788800	3.83002000
C	0.82741300	-1.05843300	3.12014500
C	2.03567700	-1.42757400	3.74160100
C	2.04644800	-2.00215000	5.01256200
C	0.84811200	-2.20190600	5.70225500
H	-1.29416700	-1.96915700	5.63704000
H	-1.31135600	-0.97839500	3.37373300
H	2.97763100	-1.26538200	3.22186100
H	2.99162300	-2.28926000	5.46479800

H	0.85445900	-2.64701400	6.69291000
C	0.87506300	-0.36121300	1.77097100
C	1.80445500	-0.81930700	0.69190000
S	2.48238700	-2.28141600	-1.52141300
O	2.21748200	-3.71456100	-1.74470000
O	1.78994400	-1.24035500	-2.29662900
C	4.24565000	-2.02372500	-1.54575300
C	5.07935800	-3.12342800	-1.31797900
C	4.75997200	-0.76643600	-1.86957800
C	6.45751100	-2.94217500	-1.39258000
H	4.65845900	-4.10035700	-1.10800400
C	6.14177400	-0.61358400	-1.94276700
H	4.09551000	0.06976200	-2.04078500
C	7.01041900	-1.69056200	-1.70675600
H	7.11463300	-3.78944900	-1.21563800
H	6.54966000	0.36329300	-2.18832600
C	8.50466700	-1.51494800	-1.82222200
H	9.04406500	-2.23597000	-1.20017500
H	8.83336600	-1.66662900	-2.85879800
H	8.81358700	-0.50680600	-1.52824800
N	1.95040500	-2.07523500	0.19075600
C	1.45381200	-3.30062300	0.85992500
H	2.12178100	-4.10358100	0.53811600
H	1.59102300	-3.16852000	1.93350400
Au	-1.02134500	-0.54080300	0.64058500
P	-3.01436800	-0.63398300	-0.63160400
C	-2.72589500	-0.05137500	-2.35283100
C	-3.75300900	0.53358900	-3.11151800
C	-1.45233800	-0.22010800	-2.92044100
C	-3.50515100	0.94048800	-4.42261400
H	-4.73958300	0.67826000	-2.68221400
C	-1.21404400	0.18561900	-4.23463000
H	-0.63849100	-0.65852600	-2.35000800
C	-2.23773300	0.76646000	-4.98533800
H	-4.30283600	1.39443000	-5.00383400
H	-0.22401400	0.05132100	-4.65947100
H	-2.04889200	1.08709200	-6.00595000
C	-3.73933700	-2.32274800	-0.76209300
C	-4.19734900	-2.95138200	0.40857900
C	-3.82276500	-2.99881400	-1.98662500
C	-4.74318500	-4.23258900	0.34845400
H	-4.13526700	-2.43774700	1.36480800
C	-4.36040100	-4.28782900	-2.03853300
H	-3.47389600	-2.52522700	-2.89841400

C	-4.82275100	-4.90427800	-0.87581800
H	-5.10528500	-4.70740000	1.25601500
H	-4.41998400	-4.80499200	-2.99173800
H	-5.24447200	-5.90420300	-0.92031200
C	-4.36502700	0.41004200	0.06353500
C	-5.71740300	0.09049700	-0.13708600
C	-4.03114700	1.56872500	0.78202400
C	-6.71726100	0.92342700	0.36804300
H	-5.99102700	-0.81002900	-0.67802700
C	-5.03450300	2.39830300	1.28339500
H	-2.98609900	1.81682700	0.94821700
C	-6.37816700	2.07719600	1.07751600
H	-7.76082400	0.66713100	0.20981600
H	-4.76838900	3.29151400	1.84194200
H	-7.15852900	2.72058900	1.47368000
C	0.03233700	-3.68727500	0.54058200
C	-0.84170900	-4.08041500	1.47021900
H	-0.22627300	-3.72836400	-0.51522000
H	-1.83103300	-4.43565200	1.19959200
H	-0.59597200	-4.07072900	2.52976300
C	2.35160600	1.26171200	0.88668400
C	3.05305200	2.50098600	0.88451000
C	2.91238900	3.37066800	1.95312100
C	2.04385700	3.09346800	3.03522300
C	1.28090000	1.94299500	3.03318600
C	1.29269400	1.09537100	1.87507300
H	3.51232300	4.27400800	1.95076600
H	2.00532300	3.78234500	3.87255900
H	0.61898900	1.70415600	3.86136400
H	0.40103100	1.91416300	1.19397700
O	3.86478600	2.32102400	-1.26839900
N	2.55231300	0.16776100	0.14670200
N	4.00317200	2.86204700	-0.17298700
O	4.85136100	3.71397700	0.09158700
C	-0.36470900	4.10049000	0.39500700
C	-0.17601300	4.60063400	-0.91813100
C	-0.16459600	5.97780900	-1.23859300
C	-0.35983200	6.83769600	-0.18719600
C	-0.56062600	6.35216600	1.14733000
C	-0.56696700	5.01341100	1.46254500
H	-0.01233200	6.30818500	-2.25968500
H	-0.36759500	7.90848300	-0.36063900
H	-0.71563600	7.07880400	1.93913700
H	-0.71796500	4.65920900	2.47520400

O	-0.12810900	2.36219300	-0.91971600
N	-0.34924000	2.75446200	0.39006000
N	-0.00867300	3.53603100	-1.73990600
O	0.17091300	3.40549700	-2.92596600

7

SCF done: -2729.891073

C	-0.51601800	-5.36375600	-0.17152800
C	-0.52168200	-4.00786700	0.16088200
C	0.64516400	-3.23272800	0.05860800
C	1.82318300	-3.86403700	-0.37784700
C	1.82572100	-5.21558100	-0.72343400
C	0.65646300	-5.97165400	-0.62167000
H	-1.43089700	-5.94258200	-0.08125800
H	-1.44208900	-3.54594200	0.50887100
H	2.74725200	-3.29641500	-0.44519800
H	2.74737700	-5.68032900	-1.06226500
H	0.66079100	-7.02493400	-0.88607600
C	0.67749300	-1.79165800	0.50915000
C	1.58641500	-0.81455800	-0.07782700
S	3.45848400	0.24489600	-1.78965100
O	3.45758700	0.24248700	-3.25698300
O	3.39142900	1.49275300	-1.00338500
C	4.80617000	-0.75330300	-1.18354800
C	5.28721100	-1.78881900	-1.99344700
C	5.39118800	-0.46088200	0.05266300
C	6.35559500	-2.55434900	-1.53703600
H	4.85014500	-1.97384100	-2.96883500
C	6.45994700	-1.24269600	0.48560100
H	5.03161000	0.36356300	0.65795400
C	6.95913300	-2.29646800	-0.29483100
H	6.73813400	-3.35698200	-2.16164000
H	6.92161600	-1.02109900	1.44390400
C	8.14230300	-3.10813100	0.17050800
H	8.11130500	-4.12783100	-0.22544400
H	9.08045000	-2.65293900	-0.17286500
H	8.18745500	-3.16295100	1.26247900
N	2.00701900	-0.69993900	-1.37467800
C	1.22872200	-1.25353000	-2.51888200
H	1.95762500	-1.52621200	-3.28505900
H	0.74434100	-2.17129600	-2.18455300
Au	-1.28973600	-0.74898500	0.13853400
P	-3.47998900	0.08428000	-0.05318400
C	-3.59886200	1.88525500	-0.39145200

C	-4.60499100	2.67065700	0.19294100
C	-2.68980500	2.46922400	-1.28746200
C	-4.70223700	4.02590400	-0.12701200
H	-5.30678700	2.23110700	0.89467700
C	-2.79680900	3.82295200	-1.60549600
H	-1.89511100	1.87852900	-1.73178300
C	-3.80220700	4.60119600	-1.02670400
H	-5.48099500	4.63029600	0.32909400
H	-2.08322500	4.26022500	-2.29697700
H	-3.88159800	5.65650800	-1.27206100
C	-4.40971700	-0.76178000	-1.39863500
C	-4.20060000	-2.13378200	-1.61523000
C	-5.33670800	-0.07076300	-2.19275000
C	-4.91599800	-2.80554100	-2.60571300
H	-3.47680000	-2.67580700	-1.01201200
C	-6.04701200	-0.74797300	-3.18658000
H	-5.50285700	0.99084100	-2.04062300
C	-5.83941100	-2.11262800	-3.39343800
H	-4.74912300	-3.86675500	-2.76640400
H	-6.76117800	-0.20569500	-3.79938700
H	-6.39213100	-2.63513100	-4.16880400
C	-4.42085500	-0.22262400	1.49934600
C	-5.75351600	-0.65913100	1.48172900
C	-3.78419300	0.01257000	2.72936800
C	-6.43904900	-0.85710400	2.68254200
H	-6.25585500	-0.84760800	0.53830600
C	-4.47670500	-0.17957300	3.92398900
H	-2.74955000	0.34586400	2.75269300
C	-5.80385500	-0.61732000	3.90196500
H	-7.46976400	-1.19898300	2.66183100
H	-3.97922600	0.00773200	4.87120500
H	-6.33992200	-0.77304200	4.83371700
C	0.23217100	-0.27169600	-3.07761400
C	-1.02618500	-0.60551600	-3.37361900
H	0.59996900	0.73175600	-3.28287400
H	-1.70566000	0.10405500	-3.83693100
H	-1.41819500	-1.60351200	-3.19007700
C	1.62357700	-0.39607800	2.14123900
C	1.87011600	0.13460000	3.41152300
C	1.30597100	-0.48573600	4.52833600
C	0.52050700	-1.62939200	4.36622700
C	0.25372900	-2.15126200	3.09596200
C	0.78912800	-1.51586600	1.97396400
H	1.50520600	-0.06998200	5.50838800

H	0.11179800	−2.11947400	5.24392900
H	−0.36073300	−3.03910300	2.98384400
H	2.53827700	0.88967000	0.73073600
O	3.33563100	1.71257100	2.58843800
N	2.07694500	−0.00274600	0.89345400
N	2.72271800	1.30057400	3.58635400
O	2.79699200	1.80956400	4.69869400
C	1.89892100	4.06777500	0.41417300
C	1.72343200	4.43308900	−0.95394800
C	2.37100200	5.54596400	−1.54678200
C	3.19326900	6.26832000	−0.72328000
C	3.39417600	5.90924600	0.65781100
C	2.77566800	4.83551500	1.23937100
H	2.21191800	5.78902300	−2.59106100
H	3.71834200	7.13371000	−1.11497000
H	4.07046100	6.51776600	1.25059100
H	2.94381400	4.54627300	2.27030200
O	0.52931800	2.63535600	−0.43051200
N	1.18266800	2.99484500	0.72457500
N	0.88102000	3.54054300	−1.49126000
O	0.38963000	3.36539200	−2.60616100

8

SCF done: -2238.965246

C	−0.06599000	4.64375500	−2.36508500
C	0.18091700	3.43640000	−1.74080400
C	−0.89153800	2.54056300	−1.42896700
C	−2.22608100	2.93039100	−1.77278800
C	−2.45995200	4.14415500	−2.39743700
C	−1.38651900	4.99567300	−2.69473200
H	0.75062700	5.31848900	−2.60071100
H	1.19348800	3.14532100	−1.47836100
H	−3.05684400	2.28069000	−1.52188500
H	−3.47306900	4.43600100	−2.65476600
H	−1.57804400	5.94599000	−3.18581600
C	−0.61355800	1.29417800	−0.82785500
C	−1.71596000	0.36157400	−0.45123900
S	−1.89874200	−2.35173100	−0.27706400
O	−1.41803800	−3.35618700	−1.24286600
O	−1.21994800	−2.12295800	1.01337200
C	−3.64030400	−2.61882400	0.00601900
C	−4.38487600	−3.27621200	−0.97688900
C	−4.21347700	−2.22748000	1.21800900
C	−5.73542000	−3.51979100	−0.74126400

H	-3.91276400	-3.60237700	-1.89676300
C	-5.56424500	-2.48622900	1.43195500
H	-3.60954100	-1.74084900	1.97422600
C	-6.34776600	-3.12634100	0.45860300
H	-6.32133800	-4.03422800	-1.49808100
H	-6.01823100	-2.19165800	2.37436300
C	-7.81796100	-3.37098900	0.69282300
H	-8.02806100	-3.56809200	1.74888300
H	-8.40793300	-2.49185900	0.40208100
H	-8.18279300	-4.21931700	0.10587400
N	-1.74320900	-0.83660000	-1.16069200
C	-1.44039400	-0.86514600	-2.59995600
H	-0.57513500	-0.21054700	-2.78832500
H	-1.09522200	-1.87489300	-2.83986200
Au	1.23918100	0.53560900	-0.35352900
P	3.34270700	-0.43385100	0.21936900
C	3.24676200	-1.40631100	1.77291900
C	4.33756400	-1.47987300	2.65359200
C	2.06367200	-2.10610800	2.06306400
C	4.24391000	-2.25143200	3.81260000
H	5.25242400	-0.93564600	2.44003700
C	1.98131600	-2.87715600	3.22300200
H	1.20424900	-2.05507000	1.39989700
C	3.06806100	-2.94999900	4.09745500
H	5.08920400	-2.30444600	4.49260000
H	1.06292300	-3.41414400	3.44056000
H	2.99840600	-3.54749800	5.00195500
C	3.93848300	-1.56476000	-1.10223900
C	3.80920000	-1.16286100	-2.44230600
C	4.51967100	-2.80517300	-0.80340500
C	4.26477700	-1.98900600	-3.46842500
H	3.35536400	-0.20424500	-2.68296300
C	4.96841900	-3.63073500	-1.83651300
H	4.61728800	-3.13035300	0.22732500
C	4.84283600	-3.22510900	-3.16607400
H	4.16357700	-1.67174000	-4.50226700
H	5.41347200	-4.59255200	-1.59930500
H	5.19067000	-3.87166300	-3.96643900
C	4.65301600	0.83425300	0.46489000
C	5.95199000	0.66445400	-0.03495900
C	4.33617300	1.99313500	1.19298600
C	6.92132400	1.64380800	0.19447500
H	6.20781300	-0.22417900	-0.60332500
C	5.30946600	2.96394400	1.42391100

H	3.33079900	2.13318600	1.58356900
C	6.60289200	2.79093500	0.92286700
H	7.92510900	1.50728200	-0.19716400
H	5.05866300	3.85576300	1.99106900
H	7.35928200	3.55023200	1.09903800
C	-2.59100900	-0.47306700	-3.49096500
C	-3.83162600	-0.17674500	-3.09928000
H	-2.33778300	-0.45779900	-4.55145700
H	-4.59677700	0.08243000	-3.82512200
H	-4.13446600	-0.19337800	-2.05632300
C	-2.47760600	1.56645700	1.47058500
C	-3.62659200	2.34051400	1.75577400
C	-3.64809600	3.26763200	2.80184400
C	-2.52188300	3.45048200	3.59360400
C	-1.38050700	2.68019700	3.34596400
C	-1.36170900	1.74977100	2.31184400
H	-4.55821000	3.83008700	2.97139900
H	-2.53648600	4.17606100	4.40001200
H	-0.50002800	2.79615500	3.97165200
H	-0.49619300	1.11233100	2.16220200
O	-4.76687300	1.72478400	-0.17374300
N	-2.51173900	0.53653700	0.53757300
N	-4.85025200	2.20444400	0.96373700
O	-5.90243500	2.58443800	1.47117600

TS8-9

SCF done:-2238.947685

C	-0.13393200	4.59112100	-1.91021500
C	0.10709000	3.22771200	-1.75834900
C	-0.95707700	2.31455400	-1.60397700
C	-2.27746400	2.81692900	-1.62224900
C	-2.51312800	4.18078800	-1.75937800
C	-1.44505200	5.07208400	-1.90729800
H	0.69999700	5.27644000	-2.02862300
H	1.12986000	2.86078000	-1.76472700
H	-3.11785000	2.13886900	-1.51072600
H	-3.53431500	4.55026900	-1.75907900
H	-1.63521700	6.13517900	-2.02277200
C	-0.67213300	0.86832800	-1.44612600
C	-1.62828300	-0.06703300	-0.74943100
S	-2.20023600	-2.73544000	-0.30627300
O	-2.02080400	-3.84998100	-1.25475700
O	-1.45194200	-2.68185300	0.96348200
C	-3.92946800	-2.44854400	-0.01963200

C	-4.82540300	-2.61177700	-1.07761400
C	-4.36002000	-2.09387800	1.26073200
C	-6.17501900	-2.35597700	-0.85153300
H	-4.47529300	-2.93092600	-2.05353700
C	-5.71498800	-1.86104000	1.46631100
H	-3.64163500	-1.99430200	2.06597700
C	-6.63788000	-1.95758600	0.41174400
H	-6.88121300	-2.46254600	-1.67019200
H	-6.06234400	-1.57635000	2.45559800
C	-8.08454700	-1.59908400	0.63376100
H	-8.45850200	-2.00294000	1.58070800
H	-8.18937200	-0.50744500	0.67915100
H	-8.72347200	-1.96680100	-0.17430600
N	-1.68262400	-1.35475900	-1.30189500
C	-0.70107400	-1.69905000	-2.33755400
H	0.22435900	-2.10455100	-1.91009400
H	-1.12545900	-2.46928300	-2.98905200
Au	1.28104200	0.34741000	-0.70820800
P	3.29017500	-0.31379600	0.33965800
C	2.93350400	-1.18553600	1.91495100
C	3.84592000	-1.16106800	2.98215800
C	1.73569700	-1.90899800	2.03812300
C	3.56020200	-1.85919900	4.15592800
H	4.76994500	-0.59667600	2.90184500
C	1.45888200	-2.60637800	3.21415700
H	1.00878100	-1.93554200	1.23122400
C	2.36952200	-2.58156000	4.27265800
H	4.26720600	-1.83548900	4.98008200
H	0.52765300	-3.15859900	3.29461000
H	2.15104900	-3.12058300	5.19005200
C	4.23743400	-1.47112400	-0.73145400
C	4.52416700	-1.08060800	-2.05117800
C	4.66410300	-2.72420200	-0.27142200
C	5.23724800	-1.93125100	-2.89370100
H	4.19872900	-0.10899900	-2.41634700
C	5.37202300	-3.57526700	-1.12407800
H	4.44611700	-3.03895600	0.74387200
C	5.65956000	-3.18166000	-2.43133500
H	5.46169000	-1.62077300	-3.91011000
H	5.69770600	-4.54603500	-0.76220400
H	6.21019200	-3.84624800	-3.09062000
C	4.41501000	1.08613700	0.72557200
C	5.80910300	0.95211100	0.64041100
C	3.85934600	2.30536600	1.14616400

C	6.63445100	2.02666400	0.97776800
H	6.25019300	0.01783200	0.30733500
C	4.68917400	3.37322600	1.48507300
H	2.77978800	2.41888200	1.20952000
C	6.07720000	3.23498400	1.40017200
H	7.71278500	1.91796600	0.90796000
H	4.25336500	4.31345500	1.81028800
H	6.72255400	4.06919300	1.65940400
C	-0.40399900	-0.45065000	-3.11782300
C	-1.38556300	0.43076300	-3.47469500
H	0.60266900	-0.32093600	-3.50282300
H	-1.15612500	1.30103900	-4.07861200
H	-2.42903400	0.25014800	-3.23837100
C	-2.42158800	1.26683900	1.12006500
C	-3.67958400	1.84983500	1.41124900
C	-3.82870800	2.83443000	2.39213200
C	-2.72327800	3.27822800	3.10520100
C	-1.47119600	2.70761200	2.84757600
C	-1.32521200	1.71618900	1.88414000
H	-4.81875200	3.23574600	2.57319100
H	-2.83558800	4.05060800	3.85872600
H	-0.60159400	3.03086700	3.41302900
H	-0.36207500	1.24020700	1.72677100
O	-4.76315400	0.99865100	-0.45696900
N	-2.31758300	0.16312800	0.29843200
N	-4.88349800	1.44153400	0.69110600
O	-5.96438700	1.56833100	1.26715100

9

SCF done: -2239.012161

C	1.48069500	2.64166100	-2.64859300
C	0.46212600	1.65705400	-2.70163800
C	-0.81606200	1.91094300	-2.13413500
C	-1.03689600	3.15151700	-1.53503400
C	-0.02008600	4.11482900	-1.46116100
C	1.23786300	3.86672900	-2.00065900
H	2.40641400	2.49033400	-3.19777600
H	0.59443900	0.78209400	-3.33455500
H	-2.01872700	3.37340100	-1.13073600
H	-0.22746000	5.07118400	-0.99037300
H	2.01481100	4.62393500	-1.96676100
C	-1.90216600	0.88546300	-2.23435900
C	-2.19761300	-0.03636600	-1.06389400
S	-3.08144400	-2.63829300	-0.64810400

O	-3.43611700	-3.61519500	-1.69172400
O	-2.03377800	-2.91979000	0.34860800
C	-4.55486400	-2.05304900	0.16352000
C	-5.75220200	-2.03833100	-0.55613800
C	-4.49303100	-1.61256200	1.48756700
C	-6.89423200	-1.52942100	0.05597900
H	-5.78905300	-2.42619600	-1.56854300
C	-5.64970800	-1.11708700	2.08190700
H	-3.55535700	-1.65322300	2.02838500
C	-6.85898300	-1.04436800	1.37224000
H	-7.82893200	-1.50583700	-0.49738800
H	-5.61272400	-0.76671100	3.10992300
C	-8.07904600	-0.42394600	2.00409800
H	-8.17341200	-0.70480800	3.05838700
H	-8.00393000	0.67038000	1.96178100
H	-8.99708000	-0.71677700	1.48621800
N	-2.56193400	-1.26538400	-1.59426700
C	-2.42650700	-1.38261000	-3.05728800
H	-1.60773500	-2.06268500	-3.31655700
H	-3.34584600	-1.77926500	-3.49473400
Au	1.92451500	0.78465700	-0.97420600
P	3.20717800	-0.54142900	0.44937100
C	2.25121400	-1.30190200	1.81245900
C	2.77578800	-1.35362400	3.11410100
C	1.00178200	-1.88297800	1.53607300
C	2.05321000	-1.98665900	4.12631700
H	3.73894000	-0.90716100	3.33869600
C	0.28969300	-2.51857800	2.55280000
H	0.57202900	-1.84808800	0.53869900
C	0.81505200	-2.56937600	3.84667900
H	2.46079100	-2.02391800	5.13232900
H	-0.67204900	-2.96101000	2.31771800
H	0.25754200	-3.06124800	4.63862300
C	3.98677000	-1.89892500	-0.50859400
C	4.58868900	-1.60634200	-1.74435300
C	4.01138300	-3.21134100	-0.01533200
C	5.21061800	-2.61720500	-2.47418500
H	4.57658600	-0.59079900	-2.13298800
C	4.63223400	-4.22001600	-0.75563500
H	3.54740000	-3.44945800	0.93594100
C	5.23019100	-3.92574400	-1.98158500
H	5.67549700	-2.38580600	-3.42798400
H	4.64441200	-5.23564500	-0.37159100
H	5.70927000	-4.71369600	-2.55519800

C	4.56023100	0.45137300	1.19173700
C	5.83953900	-0.09620600	1.37133700
C	4.29683300	1.76332800	1.61795300
C	6.84243300	0.66511100	1.97480200
H	6.05517800	-1.10703100	1.04000300
C	5.30201000	2.51551600	2.22369900
H	3.30929800	2.19532100	1.47520800
C	6.57557900	1.96746000	2.40085500
H	7.83209600	0.23877800	2.10913900
H	5.09416600	3.52987800	2.55129700
H	7.35937900	2.55722500	2.86699200
C	-2.12620200	0.04274500	-3.48476900
C	-3.11485300	1.10993900	-3.13205100
H	-1.50051900	0.18387900	-4.36082800
H	-3.09972400	2.01440800	-3.73365700
H	-4.08978800	0.84175700	-2.73853100
C	-2.00203300	1.29344400	0.94783400
C	-3.06279100	2.16347200	1.31059300
C	-2.89317500	3.19483200	2.24243600
C	-1.65512900	3.41364900	2.82820600
C	-0.59455000	2.55633900	2.50798800
C	-0.77223000	1.51268000	1.60945700
H	-3.75126500	3.80992300	2.48704600
H	-1.52247400	4.22338900	3.53810200
H	0.37368900	2.69041900	2.98316600
H	0.03180700	0.81119500	1.41416300
O	-4.50669200	1.57321100	-0.40106100
N	-2.15914100	0.14354800	0.19835900
N	-4.40349300	2.01517200	0.74663400
O	-5.35796200	2.35877700	1.44358600

TS5-10

SCF done: -2238.957366

C	-0.09791100	4.85595900	-1.92596200
C	0.12248800	3.58391200	-1.42822800
C	-0.95750800	2.66239600	-1.26618700
C	-2.27052100	3.08376000	-1.64500700
C	-2.47909100	4.35720700	-2.14563500
C	-1.39836500	5.24171100	-2.28588900
H	0.72742900	5.55114000	-2.04117200
H	1.12205600	3.26770800	-1.14584600
H	-3.10595700	2.40095000	-1.53573300
H	-3.47777000	4.67103500	-2.43255000
H	-1.57048300	6.23887700	-2.68191400

C	-0.69865100	1.36704000	-0.75084000
C	-1.80451300	0.42534500	-0.42696100
S	-1.68449900	-2.23477400	0.10224100
O	-0.80951100	-3.20282700	-0.58520700
O	-1.33596700	-1.69752500	1.43192900
C	-3.34865200	-2.88516300	0.10678300
C	-3.62082800	-4.02196100	-0.65693300
C	-4.33522500	-2.27686400	0.89016200
C	-4.91392300	-4.54473500	-0.64386700
H	-2.83188800	-4.49529900	-1.23064100
C	-5.61562000	-2.81761100	0.88735400
H	-4.09962200	-1.39927900	1.47985200
C	-5.92864400	-3.95430900	0.12074200
H	-5.13324100	-5.43122200	-1.23252400
H	-6.38896600	-2.35235100	1.49304600
C	-7.32868300	-4.51685500	0.12812500
H	-8.04039900	-3.80283600	-0.30439400
H	-7.39306200	-5.44495000	-0.44672300
H	-7.66504700	-4.72645400	1.15015100
N	-1.72076500	-0.85612200	-0.98089900
C	-1.48424500	-1.17896500	-2.41872100
H	-0.50652600	-1.65892400	-2.51674400
H	-2.24484300	-1.91453100	-2.70188900
Au	1.15132400	0.58106200	-0.31161400
P	3.25522500	-0.41295600	0.22179700
C	3.24096200	-1.23582900	1.86318600
C	4.39853200	-1.29688800	2.65568800
C	2.05160800	-1.83115900	2.31486000
C	4.36424400	-1.95397000	3.88616400
H	5.31965000	-0.83109100	2.31901500
C	2.02819700	-2.48731300	3.54602700
H	1.14290700	-1.78769800	1.72130300
C	3.18126500	-2.54899000	4.33158000
H	5.26127100	-1.99775900	4.49705000
H	1.10425800	-2.94360300	3.88851000
H	3.15824400	-3.05644700	5.29177300
C	3.71841800	-1.68010700	-1.02725100
C	3.53972600	-1.37998600	-2.38830500
C	4.24192700	-2.92591500	-0.65514400
C	3.89104700	-2.31236000	-3.36304200
H	3.13070000	-0.41713300	-2.68601800
C	4.58564500	-3.85822000	-1.63656200
H	4.37588300	-3.17232000	0.39311700
C	4.41223200	-3.55376100	-2.98742800

H	3.75337200	-2.07307800	-4.41355400
H	4.98660700	-4.82354800	-1.34187200
H	4.67888200	-4.28263100	-3.74722600
C	4.62350100	0.81702900	0.25886600
C	5.87950700	0.54461200	-0.30151300
C	4.39895300	2.05220700	0.88935100
C	6.89748500	1.49852200	-0.22969700
H	6.06414300	-0.40449500	-0.79469700
C	5.42059800	2.99771600	0.96323500
H	3.42792000	2.27084800	1.32809600
C	6.67065900	2.72241600	0.40150700
H	7.86752000	1.28203900	-0.66765300
H	5.24136700	3.94902000	1.45605900
H	7.46466900	3.46157400	0.45519700
C	-1.57896900	0.00597200	-3.34031800
C	-2.63406800	0.25076600	-4.11994000
H	-0.70291500	0.64927400	-3.40269500
H	-2.64969500	1.08921300	-4.81077900
H	-3.51769000	-0.38324300	-4.10427800
C	-2.74900400	1.70817400	1.36124400
C	-3.93252000	2.45736600	1.55222000
C	-4.03780800	3.42048700	2.55926800
C	-2.96472600	3.65813600	3.40889000
C	-1.79130700	2.91188400	3.25450300
C	-1.68619200	1.94941700	2.25558300
H	-4.96858000	3.96668300	2.65260200
H	-3.04453300	4.40934300	4.18752300
H	-0.95214700	3.07316000	3.92522200
H	-0.80085100	1.32703100	2.17854400
O	-4.93079600	1.66573700	-0.38674300
N	-2.69734400	0.64216800	0.47241000
N	-5.09524600	2.25771800	0.68784900
O	-6.17751600	2.69274400	1.07371500

10

SCF done:-2238.964066

C	-0.08286100	4.58057500	-2.54053500
C	0.13359600	3.39647500	-1.85940900
C	-0.95721500	2.53659600	-1.52316500
C	-2.27780400	2.93518400	-1.90112500
C	-2.48327800	4.12378300	-2.58140800
C	-1.39039800	4.94226300	-2.90344600
H	0.75006000	5.22830500	-2.79441500
H	1.13724000	3.09942700	-1.57096000

H	-3.12577900	2.31496000	-1.62800500
H	-3.48810100	4.42248500	-2.86268300
H	-1.55817300	5.87244700	-3.43955000
C	-0.71093400	1.31632300	-0.84816100
C	-1.83838100	0.42515900	-0.45520700
S	-1.93551100	-2.29261200	-0.21388000
O	-1.31014200	-3.27148200	-1.12281300
O	-1.35794000	-1.96571000	1.10360200
C	-3.66188100	-2.71322200	-0.03825700
C	-4.21419900	-3.64545400	-0.92008900
C	-4.41779700	-2.14522800	0.99188900
C	-5.55664900	-3.99157600	-0.77567300
H	-3.59903300	-4.10675700	-1.68456100
C	-5.75393400	-2.50909500	1.11709500
H	-3.96426900	-1.43698000	1.67431100
C	-6.34762800	-3.42883000	0.23553700
H	-5.99247300	-4.71977600	-1.45418500
H	-6.34904200	-2.07586400	1.91668400
C	-7.80594100	-3.78981800	0.37410500
H	-8.44377700	-2.98753700	-0.01885100
H	-8.04837900	-4.70427800	-0.17491400
H	-8.08236800	-3.93684800	1.42365000
N	-1.87895800	-0.80168700	-1.12712900
C	-1.78770600	-0.88938300	-2.60412300
H	-2.35842000	-1.77730100	-2.89414900
H	-2.31669400	-0.02775300	-3.02431700
Au	1.11646700	0.54473300	-0.31583200
P	3.19074600	-0.42648100	0.34892800
C	3.06654500	-1.27443000	1.97356500
C	4.14029500	-1.28630700	2.87739900
C	1.87196600	-1.93986400	2.29629400
C	4.01852100	-1.96309000	4.09193900
H	5.06388000	-0.76778000	2.63945900
C	1.76179900	-2.61671400	3.51134900
H	1.02576600	-1.93361100	1.61450500
C	2.83189600	-2.62829600	4.40911700
H	4.85077100	-1.96802700	4.78986200
H	0.83500100	-3.12807900	3.75379700
H	2.74059800	-3.15163000	5.35663800
C	3.75769600	-1.67855600	-0.87439200
C	3.67600300	-1.36635500	-2.24192400
C	4.26995800	-2.92114200	-0.47644600
C	4.11221300	-2.28364900	-3.19657300
H	3.27339700	-0.40702300	-2.55854200

C	4.69884100	-3.83846900	-1.43856400
H	4.32964300	-3.17653300	0.57655900
C	4.62188800	-3.52197300	-2.79550900
H	4.05106600	-2.03547700	-4.25235600
H	5.09097300	-4.80122800	-1.12422800
H	4.95470900	-4.23884000	-3.54044700
C	4.54283000	0.81299200	0.49076400
C	5.86016400	0.51245400	0.11402300
C	4.24472300	2.08155100	1.01469200
C	6.86507600	1.47041300	0.26434100
H	6.10200100	-0.46189800	-0.29892200
C	5.25318100	3.03245500	1.16660900
H	3.22565300	2.32353100	1.30715800
C	6.56409200	2.72810900	0.79015400
H	7.88258600	1.23204700	-0.03141800
H	5.01580200	4.01068600	1.57484600
H	7.34813900	3.47107100	0.90410300
C	-0.38769100	-0.98539600	-3.15240300
C	0.07510500	-0.20467100	-4.13203200
H	0.22496000	-1.78180500	-2.73578100
H	1.07080000	-0.33985200	-4.54489900
H	-0.52975300	0.58438100	-4.57499000
C	-2.59684600	1.71298800	1.40330900
C	-3.71915100	2.54176200	1.63245900
C	-3.72202800	3.51166600	2.63893200
C	-2.60505300	3.67950200	3.44735200
C	-1.49202600	2.85424900	3.25549700
C	-1.49059300	1.88317800	2.25876000
H	-4.61058600	4.11815400	2.76505100
H	-2.60569400	4.43710300	4.22390400
H	-0.62028500	2.95901100	3.89517000
H	-0.64978600	1.20572700	2.15209700
O	-4.85054900	1.85602900	-0.27800700
N	-2.64991300	0.64451400	0.51041200
N	-4.93080200	2.42240100	0.81885000
O	-5.96949100	2.89829400	1.27020700

TS10-11

SCF done: -2238.939402

C	0.12958400	4.86222300	-1.40785000
C	0.27764700	3.48139600	-1.31689500
C	-0.83682800	2.62146000	-1.42214200
C	-2.10578100	3.20076100	-1.65113900
C	-2.25214800	4.58133400	-1.73514400

C	-1.13638300	5.41690400	-1.61620900
H	0.99941300	5.50610200	-1.31772100
H	1.26478900	3.05535500	-1.15941800
H	-2.98413500	2.56839500	-1.73442300
H	-3.23752300	5.00802500	-1.89787500
H	-1.25317600	6.49430400	-1.68950800
C	-0.65122500	1.14849000	-1.37597400
C	-1.78663000	0.29575100	-0.84926400
S	-2.01112700	-2.44960200	-0.40375100
O	-1.58182700	-3.51479700	-1.32895500
O	-1.26363300	-2.10993800	0.81734000
C	-3.73837300	-2.66642900	-0.03176600
C	-4.50431100	-3.48854400	-0.86377800
C	-4.28857900	-2.05181300	1.09809700
C	-5.85225500	-3.67178900	-0.56521100
H	-4.04910400	-3.99523700	-1.70748400
C	-5.63541700	-2.25452600	1.37626900
H	-3.67093500	-1.43741300	1.74095500
C	-6.44110300	-3.05557100	0.54909000
H	-6.45398400	-4.31355300	-1.20264200
H	-6.07156400	-1.78493900	2.25378700
C	-7.90715100	-3.23674900	0.85252100
H	-8.47544500	-2.34509500	0.55782600
H	-8.32768600	-4.09093400	0.31426200
H	-8.07689800	-3.38799100	1.92384400
N	-1.90012200	-1.00909000	-1.41902900
C	-2.03984300	-1.14154600	-2.87684400
H	-2.14695200	-2.19601200	-3.12948600
H	-2.87792300	-0.55585700	-3.26627100
Au	1.16220200	0.36953800	-0.55170900
C	3.95735100	-1.67285900	-0.83460800
C	4.11921300	-1.28836000	-2.17708700
C	4.36145600	-2.95157000	-0.42866200
C	4.68647800	-2.16974000	-3.09548000
H	3.81123700	-0.29638000	-2.50035900
C	4.92124900	-3.83410000	-1.35626000
H	4.23910700	-3.26203400	0.60380200
C	5.08527600	-3.44607700	-2.68619200
H	4.81644300	-1.86290500	-4.12944300
H	5.22938500	-4.82469400	-1.03482600
H	5.52209100	-4.13448000	-3.40379700
C	4.43509800	0.85885300	0.59051800
C	5.79955500	0.65544600	0.33546000
C	4.00088300	2.09967700	1.08471900

C	6.71546100	1.68212200	0.57494800
H	6.14739900	-0.29716900	-0.05186800
C	4.92064500	3.11945200	1.32638900
H	2.94474800	2.26625700	1.28300900
C	6.27862000	2.91215900	1.06994100
H	7.77008300	1.51873600	0.37314000
H	4.57774900	4.07591400	1.71049500
H	6.99368600	3.70885600	1.25336600
C	-0.69791600	-0.59603600	-3.19257500
C	-0.50656900	0.78408400	-3.23419200
H	0.14180500	-1.27730400	-3.07129200
H	0.48153700	1.16688400	-3.46700200
H	-1.33059300	1.37511300	-3.62705000
C	-2.53553900	1.56464300	1.05781100
C	-3.68604300	2.34050000	1.33420200
C	-3.71709600	3.25822100	2.38778900
C	-2.60259400	3.42420700	3.19912400
C	-1.46329400	2.64650600	2.96288500
C	-1.43253800	1.72938000	1.91825700
H	-4.62620500	3.82476100	2.54848300
H	-2.62411500	4.14238000	4.01205800
H	-0.59279800	2.74920000	3.60475300
H	-0.56379700	1.09658800	1.76751300
O	-4.80583400	1.71614300	-0.60275600
N	-2.56811300	0.53236100	0.13127600
N	-4.89918700	2.21043500	0.52670700
O	-5.95596500	2.60306200	1.01896400
C	3.00132700	-1.33427800	1.94545300
C	4.02783200	-1.32357100	2.90351900
C	1.81004300	-2.03109300	2.20533100
C	3.86236500	-2.00878400	4.10757700
H	4.94826500	-0.77929500	2.71618800
C	1.65583100	-2.71697500	3.41096400
H	1.00079700	-2.04022100	1.48092500
C	2.67861200	-2.70594700	4.36175700
H	4.65771500	-1.99515900	4.84715200
H	0.73128600	-3.25259800	3.60506100
H	2.55286200	-3.23580800	5.30168500
P	3.19233700	-0.47377700	0.33437700

11

SCF done: -2238.980117

C	0.60149000	4.78110500	-1.67759300
C	0.58640300	3.38715900	-1.60803200

C	-0.62280000	2.67746000	-1.52913000
C	-1.82093600	3.41235500	-1.54449600
C	-1.80641600	4.80664900	-1.60523300
C	-0.59571900	5.49779400	-1.67002700
H	1.55137000	5.30502900	-1.73964100
H	1.52793500	2.84362600	-1.62391600
H	-2.77656900	2.89727000	-1.50176200
H	-2.74637700	5.35177500	-1.60830000
H	-0.58563000	6.58275100	-1.72093400
C	-0.64301600	1.15987500	-1.56638900
C	-1.73222000	0.47286300	-0.83251300
S	-2.01117800	-2.47514300	-0.65325900
O	-1.71923500	-3.38834900	-1.76668600
O	-1.09650800	-2.22421500	0.46216500
C	-3.68465200	-2.66377700	-0.12098700
C	-4.53809400	-3.47325000	-0.88172800
C	-4.10442700	-2.05344300	1.06817100
C	-5.84669900	-3.65016600	-0.44309700
H	-4.17710800	-3.97124700	-1.77487800
C	-5.41649700	-2.24884000	1.47957200
H	-3.42166200	-1.44124000	1.64357800
C	-6.30985400	-3.03847200	0.73285100
H	-6.51718600	-4.28108100	-1.01926800
H	-5.75742700	-1.78159700	2.39905800
C	-7.73674300	-3.20977900	1.18504300
H	-8.32277200	-2.31203100	0.94917700
H	-8.21749400	-4.05954000	0.69282600
H	-7.79611300	-3.35812500	2.26827000
N	-2.03048300	-0.80387200	-1.57512900
C	-2.93069800	-0.66627900	-2.75451500
H	-3.50775900	-1.56007000	-2.97216900
H	-3.47842700	0.27166200	-2.78128700
Au	1.12012800	0.28337900	-0.61123600
C	3.78952600	-1.99610400	-0.57184900
C	3.74168800	-1.98627000	-1.97580900
C	4.34903100	-3.09689100	0.09261700
C	4.25586700	-3.05861900	-2.70307200
H	3.30647600	-1.13815100	-2.49894900
C	4.85624300	-4.17163400	-0.64135600
H	4.38730800	-3.12043200	1.17700100
C	4.81160600	-4.15399100	-2.03611600
H	4.21894500	-3.04186200	-3.78855200
H	5.28537400	-5.02229700	-0.11989800
H	5.20618700	-4.99202100	-2.60334700

C	4.44884300	0.73704600	0.30511900
C	5.78339900	0.41359900	0.01929700
C	4.10515300	2.06880500	0.59091300
C	6.76018900	1.41178000	0.02273000
H	6.06077900	-0.61051400	-0.20966500
C	5.08602600	3.05975100	0.59939700
H	3.07175900	2.33069200	0.80477300
C	6.41419000	2.73257700	0.31325400
H	7.79102000	1.15481100	-0.20326700
H	4.81271900	4.08702900	0.82273600
H	7.17626900	3.50650900	0.31312000
C	-1.46047700	-0.70015000	-2.95738500
C	-0.69826600	0.61362100	-3.03227100
H	-1.02815800	-1.62540700	-3.32463100
H	0.29005300	0.47280900	-3.47170600
H	-1.24330700	1.31514800	-3.67666400
C	-2.43813400	1.67684000	1.10452800
C	-3.61114900	2.40681200	1.40076000
C	-3.65288000	3.34204500	2.43705500
C	-2.51677900	3.57613600	3.20202500
C	-1.34885400	2.85332100	2.93593900
C	-1.31130500	1.91396000	1.90986600
H	-4.57996400	3.87233900	2.61906900
H	-2.54179900	4.31131600	3.99954900
H	-0.45891500	3.02012100	3.53603200
H	-0.41214500	1.33433600	1.72643300
O	-4.73288500	1.80964800	-0.54480400
N	-2.47035700	0.63207200	0.18353500
N	-4.83332200	2.21519900	0.61962900
O	-5.90378400	2.47417400	1.16669000
C	2.94747700	-1.05755800	2.09734000
C	3.96286700	-0.82747200	3.03814900
C	1.77025000	-1.71564700	2.49062400
C	3.80141300	-1.25508900	4.35736000
H	4.87420900	-0.31480800	2.74660300
C	1.61981200	-2.14493200	3.80932600
H	0.97554000	-1.89417100	1.77154700
C	2.63265700	-1.91408700	4.74368400
H	4.58992200	-1.07186500	5.08157000
H	0.70873300	-2.65599200	4.10716600
H	2.51017500	-2.24475400	5.77112700
P	3.12573700	-0.54221300	0.34064600

P1

SCF done:-1402.245581

C	4.99333100	-1.15685700	0.44078700
C	3.67956400	-1.58133400	0.24432700
C	2.85644400	-0.94682100	-0.69799400
C	3.37874500	0.11789900	-1.44296800
C	4.69387000	0.54326300	-1.24934800
C	5.50371200	-0.09208100	-0.30638200
H	5.62004800	-1.65964500	1.17260800
H	3.28609900	-2.41355300	0.82323900
H	2.74470400	0.61793900	-2.16971000
H	5.08442200	1.37192700	-1.83381400
H	6.52836200	0.23732500	-0.15635200
C	1.44553800	-1.40919800	-0.89771700
C	0.38790200	-1.08878500	0.13324700
S	-1.98611400	-2.17547500	1.08026500
O	-2.64768000	-3.39912900	0.58511100
O	-1.65601000	-1.99702600	2.50113700
C	-2.86830600	-0.74032400	0.48699000
C	-3.53616200	-0.81433300	-0.73630600
C	-2.85731200	0.43358900	1.24120100
C	-4.15819600	0.32906600	-1.23071000
H	-3.56656500	-1.74903900	-1.28634500
C	-3.49487200	1.56213500	0.73346000
H	-2.34435800	0.45413500	2.19522700
C	-4.12616500	1.53738200	-0.51921800
H	-4.66683400	0.28721100	-2.19035300
H	-3.48076900	2.48572500	1.30637100
C	-4.70796600	2.79882200	-1.10622700
H	-5.19203100	3.41540600	-0.34104200
H	-3.90451300	3.39860700	-1.55307300
H	-5.44157500	2.58164100	-1.88905000
N	-0.52121900	-2.14217400	0.14683600
C	-0.09780100	-3.32295200	-0.62750800
H	0.19569000	-4.13990800	0.04225300
H	-0.91954700	-3.67727200	-1.25477100
C	1.08744900	-2.79932100	-1.41887300
C	0.86296800	-1.60159200	-2.28968500
H	1.88051600	-3.49636500	-1.67026300
H	1.55468200	-1.45458700	-3.11404100
H	-0.14761500	-1.24979300	-2.47409100
C	0.96717700	1.09520300	0.91584900
C	0.64499300	2.25261300	0.16340300
C	1.28734500	3.47892300	0.38059400
C	2.28871600	3.58401400	1.33254000
C	2.62296100	2.45652700	2.09595100

C	1.96371000	1.25167100	1.90479900
H	0.98252600	4.32891000	-0.21801500
H	2.79854100	4.52979500	1.48622800
H	3.39818500	2.52392700	2.85443700
H	2.19856900	0.38734500	2.51666000
O	-0.60835200	1.17568200	-1.46485500
N	0.23231600	-0.07600600	0.89101400
N	-0.38285800	2.23373900	-0.87346100
O	-0.96723800	3.29471100	-1.11918600

P2

SCF done: -1402.273274

C	0.48155500	4.74164900	-0.19167600
C	-0.36279800	3.63602300	-0.08795600
C	0.13754500	2.32821700	-0.22924400
C	1.51243200	2.16520400	-0.47682200
C	2.35641400	3.27178200	-0.57091700
C	1.84554900	4.56425100	-0.43200900
H	0.07363200	5.74246300	-0.07640800
H	-1.41874000	3.78039700	0.12284100
H	1.91453500	1.16612700	-0.61185500
H	3.41552800	3.12243000	-0.76425300
H	2.50404400	5.42495700	-0.51086200
C	-0.76873500	1.16836400	-0.13218900
C	-0.60232900	0.01027500	0.61247400
S	1.20480400	-1.82813400	1.42162300
O	1.95943500	-1.97256400	2.68012400
O	0.19607300	-2.81729100	0.98950800
C	2.37536100	-1.63835000	0.07510800
C	3.67662900	-1.21444000	0.35401100
C	1.96939200	-1.91040900	-1.23370400
C	4.57285700	-1.04706900	-0.69981000
H	3.98083800	-1.03466300	1.37961300
C	2.88016300	-1.73279500	-2.27325800
H	0.96365500	-2.26631000	-1.42821700
C	4.19197700	-1.30102600	-2.02610800
H	5.58818400	-0.72133800	-0.48768800
H	2.56852600	-1.94118400	-3.29367100
C	5.18003600	-1.14852500	-3.15783500
H	5.95873200	-0.41829700	-2.91543500
H	5.68094600	-2.10214900	-3.37092300
H	4.68589400	-0.82809300	-4.08091100
N	0.39903400	-0.32065700	1.53719600
C	0.72206500	0.61417100	2.64616900

H	1.63749700	0.23894100	3.10853100
H	0.93112400	1.60164100	2.22525500
C	-0.38785600	0.68340600	3.65875500
C	-1.06519800	1.79729300	3.93710700
H	-0.61883900	-0.24968400	4.17145600
H	-1.85237800	1.81683800	4.68582200
H	-0.85202400	2.73523100	3.42807800
C	-2.57258900	-0.24497000	-0.43311900
C	-3.79325300	-0.68592800	-0.97341200
C	-4.48504500	0.13214200	-1.86484400
C	-3.95935000	1.37643200	-2.23163900
C	-2.74531800	1.82696700	-1.71214600
C	-2.04144500	1.02727400	-0.80169000
H	-5.42860100	-0.22166900	-2.26151400
H	-4.50752700	1.99556400	-2.93486800
H	-2.34311300	2.78814600	-2.01778500
H	-1.77943800	-1.76647700	0.79394900
O	-3.68955000	-2.66971900	0.18639400
N	-1.69994800	-0.82279900	0.43673400
N	-4.34179000	-1.97868400	-0.61037700
O	-5.41455300	-2.32509800	-1.10396400
